# Influenza A genomic diversity during human infections underscores the strength of genetic drift and the existence of tight transmission bottlenecks

**DOI:** 10.1093/ve/veae042

**Published:** 2024-06-01

**Authors:** Michael A Martin, Nick Berg, Katia Koelle

**Affiliations:** Department of Pathology, Johns Hopkins School of Medicine, 600 N. Wolfe Street, Baltimore, MD 21287, USA; Graduate Program in Population Biology, Ecology, and Evolution, Emory University, 1462 Clifton Road NE, Atlanta, GA 30322, USA; Department of Biology, Emory University, 1510 Clifton Road NE, Atlanta, GA 30322, USA; Department of Biology, Emory University, 1510 Clifton Road NE, Atlanta, GA 30322, USA; Department of Biochemistry, Brandeis University, 415 South Street, Waltham, MA 02453, USA; National Institute of Allergy and Infectious Diseases Laboratory of Viral Disease, National Institutes of Health, 33 North Drive, Bethesda, MD 20814, USA; Department of Biology, Emory University, 1510 Clifton Road NE, Atlanta, GA 30322, USA; Emory Center of Excellence for Influenza Research and Response (Emory-CEIRR), 1510 Clifton Road NE, Atlanta, GA 30322, USA

## Abstract

Influenza infections result in considerable public health and economic impacts each year. One of the contributing factors to the high annual incidence of human influenza is the virus’s ability to evade acquired immunity through continual antigenic evolution. Understanding the evolutionary forces that act within and between hosts is therefore critical to interpreting past trends in influenza virus evolution and in predicting future ones. Several studies have analyzed longitudinal patterns of influenza A virus genetic diversity in natural human infections to assess the relative contributions of selection and genetic drift on within-host evolution. However, in these natural infections, within-host viral populations harbor very few single-nucleotide variants, limiting our resolution in understanding the forces acting on these populations *in vivo*. Furthermore, low levels of within-host viral genetic diversity limit the ability to infer the extent of drift across transmission events. Here, we propose to use influenza virus genomic diversity as an alternative signal to better understand within- and between-host patterns of viral evolution. Specifically, we focus on the dynamics of defective viral genomes (DVGs), which harbor large internal deletions in one or more of influenza virus’s eight gene segments. Our longitudinal analyses of DVGs show that influenza A virus populations are highly dynamic within hosts, corroborating previous findings based on viral genetic diversity that point toward the importance of genetic drift in driving within-host viral evolution. Furthermore, our analysis of DVG populations across transmission pairs indicates that DVGs rarely appeared to be shared, indicating the presence of tight transmission bottlenecks. Our analyses demonstrate that viral genomic diversity can be used to complement analyses based on viral genetic diversity to reveal processes that drive viral evolution within and between hosts.

## Introduction

Despite relatively widespread vaccination, human influenza infections result in over 20,000 deaths and $3.7 billion in direct medical costs each year in the USA alone (Paget et al., 2019). One of the contributing factors to the virus’s widespread circulation is its ability to rapidly evolve to evade natural and vaccine-derived immunity (Koelle et al., 2006; Boni, 2008; Smith et al., 2004; Bedford et al., 2014; Bedford et al., 2015). This so-called ‘antigenic drift’ allows the virus to continually replenish its pool of susceptible hosts by reinfecting hosts who already harbor immunity to previously circulating strains. Understanding how new antigenic variants evolve within single hosts and ultimately sweep the population is important for vaccine strain selection (Neher et al., 2016; Huddleston et al., 2020; Castro et al., 2020) and informing the development of vaccines that are more robust to viral evolution (Viboud et al., 2020). The evolution of antigenically diverse lineages of influenza virus is possible because there is diversity in the viral population on which selection can act. This diversity is generated by errors made by the viral polymerase during replication within single hosts and is impacted by the evolutionary forces that occur within hosts. Population bottlenecks that occur during transmission between hosts further shape this viral diversity (McCrone and Lauring, 2018). Analyzing these dynamics can therefore provide insights into the evolution acting within and between hosts.

Deep sequencing data can be used to characterize patterns of viral genetic diversity within individual hosts. To do so, sequencing reads are aligned to a reference genome and used to estimate the frequency of each nucleotide at each site of the viral genome. By analyzing the frequencies of these intrahost single-nucleotide variants (iSNVs) across multiple time points, one can determine whether selection is acting on specific mutations or whether genetic drift dominates within-host evolution (Xue et al., 2018). Finally, by comparing iSNV frequencies in donor and recipient hosts in transmission pairs, one can estimate how many viral particles seeded infection in the recipient (Sobel Leonard et al., 2017). Previous analyses of this type have revealed that selection during an acute natural influenza A virus infection is relatively weak. Positive selection acting on known antigenic escape mutations is not apparent, and the presence of prior immunity has little impact on the amount of observed within-host genetic diversity (Debbink et al., 2017; McCrone et al., 2018). It has been suggested that this may be due to a mismatch in the timing between viral population growth and the immune response (Morris et al., 2020). While evidence for positive selection is limited, purifying selection does appear to act within these infections and contribute to shaping *in vivo* influenza virus populations (McCrone et al., 2018).

Our understanding of influenza A virus evolution within individuals and between transmission pairs stems from analyses across many individual infections and transmission pairs. This is because within any given acute infection, there is limited viral genetic diversity. At a 2 to 3 percent variant calling threshold, the number of iSNVs identified in a viral sample is generally fewer than 15, and most of these iSNVs occur at low frequencies (Debbink et al., 2017; McCrone et al., 2018). This limited genetic diversity hinders our ability to robustly characterize the evolutionary forces acting on these populations and results in considerable uncertainty in our inferred contributions of selection and drift to within- and between-host evolution. Here, we propose using an alternative signal to study the evolutionary dynamics of viral populations within and between hosts. Specifically, we propose focusing on viral genomic diversity that is generated during infection in the form of influenza defective viral genomes (DVGs).

DVGs (here used synonymously with deletion-containing viral genomes (Alnaji et al., 2021)) harbor a large internal deletion in at least one of the eight segments of the influenza A virus genome. As a result, virions with a DVG are incapable of replicating on their own. However, through coinfection of a cell with an infectious ‘wild-type’ virus, they can proliferate throughout an infection (Brooke, 2014; Genoyer and López, 2019). The process by which coinfection rescues cellular infection with DVGs is similar to the process by which coinfection can rescue infection by virions with incomplete genomes, (Jacobs et al., 2019; Brooke et al., 2013) except that instead of missing entire segments, DVGs harbor truncated copies of gene segments. Individual DVG segments can be identified by the genomic sites at which these deletions occur. We refer to these unique DVG segments as ‘DVG species’. Due to the reliance on coinfection, we expect the evolutionary forces acting on populations of DVG species to mirror those acting on the wild-type viral population. For example, if positive selection were to be acting on a specific viral mutation, then DVG species that coinfect with wild-type viruses harboring this mutation would appear to have an evolutionary advantage over the DVG species, which coinfect with viruses lacking this beneficial mutation. This is similar to the process of genetic hitchhiking, in which loci that are linked to beneficial mutations will increase in frequency (Barton et al., 2000). Hitchhiking can occur due to physical linkage on a gene segment or through spatial structure that maintains linkage disequilibrium. Given the extent of spatial structure in within-host influenza virus infections (Gallagher et al., 2018), we therefore expect linkage to be strong not only within gene segments but also between them, consistent with the limited effective reassortment between gene segments that has been found in a longitudinally sampled human influenza challenge study (Sobel Leonard et al., 2017). With linkage between DVGs and co-circulating wild-type viruses, the dynamics of DVGs may thus offer a complementary signal of evolutionary processes occurring within hosts to that which is presented by viral genetic diversity in the form of iSNVs.

It has been proposed that DVGs may also be transmitted between hosts (Saira et al., 2013). Transmission of DVGs between hosts must rely on coinfection of recipient host cells by both wild-type and DVG virions. *A priori*, this is expected to be unlikely under the assumption that wild-type and DVG virions infect host cells at random during transmission, given the large number of susceptible host cells. However, it is thought that some viruses, including influenza viruses (Wallis and Melnick, 1967), may form so-called collective infectious units (Sanjuán et al., 2017). In these collective units, virions aggregate together, presumably increasing the probability of cellular coinfection. This may play a role in how influenza viruses are able to transmit at all, given the high proportion of virions harboring incomplete viral genomes (Jacobs et al., 2019; Brooke et al., 2013). If viral aggregates form and wild-type virions within these aggregates are genetically identical due to the spatial structuring of the source individual’s viral population, then DVGs may provide more resolution into the characterization of transmission bottlenecks.

Here, we apply a recently developed bioinformatic pipeline (Alnaji et al., 2019) to previously published deep sequencing data from 217 clinical samples of influenza A H3N2 infections from 168 naturally infected, otherwise healthy, individuals from a cohort study (McCrone et al., 2018). For 49 of these 168 individuals, longitudinally sampled sequence data are available, allowing us to examine DVG dynamics within hosts over the course of their infections. Furthermore, deep sequenced virus samples are available for thirty-nine epidemiological transmission pairs, allowing us to use DVG diversity to characterize the transmission bottleneck between donors and recipients.

## Materials and methods

### Influenza virus deep sequence data

All clinical and sequencing data were previously published as part of (McCrone et al., 2018), and all epidemiological and laboratory methods are described in detail in the original publication. In short, the Household Influenza Vaccine Effectiveness cohort at the University of Michigan School of Public Health queries participating households weekly during the months of October through May for symptoms of respiratory illness. Individuals with symptoms were sampled via a combined nasal and throat swab by the research team. During the 2014–5 season, individuals were also instructed to take a self- or parent-collected nasal swab at symptom onset.

As described in McCrone et al., 2018, the amount of viral RNA in each sample was quantified through amplification of the M-segment with Centers for Disease Control and Prevention reverse transcription polymerase chain reaction (RT-PCR) primers. Cycle threshold (Ct) values were converted to genomes/*µ*l based on dilutions of a plasmid control. Complementary deoxyribonucleic acid (cDNA) was amplified from samples testing positive for influenza virus using the SuperScript III One-Step RT-PCR Platinum Taq HiFi Kit and the universal influenza A primers (Zhou et al., 2009). Sequencing libraries were prepared from 300 to 400 base pair (bp) sheared cDNA fragments, and barcoded libraries were further purified by isolation of a 300–500 bp band using gel isolation. 2 × 125 nucleotide paired end reads were generated on an Illumina HiSeq 2500. Samples with input titers between 10^3^ and 10^5^ genomes/*µ*l were sequenced in replicate. For each sequencing run, PCR amplicons derived from eight clonal plasmids of the circulating strain were sequenced on the same HiSeq flow cell as the clinical samples.

Influenza A H3N2 (as identified by a library labeled ‘perth’, ‘hk’, or ‘vic’) sequencing reads were downloaded from the National Library of Medicine (NLM) National Center for Biotechnology Information (NCBI) Sequencing Read Archive (SRA) BioProject PRJNA412631 (McCrone et al., 2018) using the fasterq-dump utility available as part of the SRA Toolkit (https://github.com/ncbi/sra-tools).

### DVG identification

DVGs were identified using a modified version of the pipeline presented in Alnaji et al. (2019). The analysis pipeline was run in Nextflow v19.01.0.5050 (Di Tommaso et al., 2017). For quality control, sequencing reads were first trimmed using Trimmomatic v0.38 (Bolger et al., 2014) in Phred33 mode using the TruSeq3-PE-2 adapters allowing for two seed mismatches, a palindromeClipThreshold of 15, and a simpleClipThreshold of 10, scanning the read with a 3 base sliding window and cutting when the average quality falls below 20, removing leading and trailing bases with quality less than 28, and removing reads less than 75 nucleotides in length. Next, Kraken2 v.2.0.7-beta (Wood et al., 2019) with the k2_pluspf_16gb database in paired-end mode was used to categorize each read. The extract_kraken_reads.py script from Kraken Tools (https://github.com/jenniferlu717/KrakenTools) was used to filter only for reads assigned to influenza A virus (taxonomic id 11320) or any children taxa. Quality control on the filtered reads was conducted with FastQc v0.11.8 (Andrews, 2010).

The paired-end reads were concatenated into a single fastq file and aligned using Bowtie2 v.2.3.4.3 (Langmead and Salzberg, 2012) in end-to-end mode with a minimum scoring scheme of L,0,−0.3. Reference genomes for this initial alignment step were used in accordance with those used in (McCrone et al., 2018): GenBank CY121496-503 was used for samples collected as part of the 2010–11 or 2011–2 season, GenBank KJ942680-8 was used for samples collected during the 2012–3 season, and GenBank CY207731-8 was used for samples collected during the 2014–5 season. End-to-end mode disallows soft-clipping of reads. Because soft-clipping is needed to align reads that span DVG junction sites, reads which do align in end-to-end mode are the subset of reads that are wild-type viral reads (as well as reads from the 5^ʹ^ and 3^ʹ^ ends of DVGs which do not span their respective deletion junction sites). We refer to this subset of reads as wild-type reads, remaining cognizant that this subset also likely contains some reads derived from DVGs. The number of wild-type reads that aligned to each position in the genome was calculated using Samtools v1.9 with htslib v.1.9 (Li et al., 2009; Li, 2011) by first sorting by name, then adding mate score tags with fixmate, sorting again by coordinates, marking and removing duplicates with markdup, and finally tabulating reads using idxstats.

Wild-type reads were then used to identify consensus variants (those present at >50 percent allele frequency) relative to the reference genome using Samtools and Bcftools v.1.8 (Li, 2011) with a maximum read depth of 1,000, minimum read quality of 20, and minimum mapping quality of 20. Consensus variants were used to generate a run-specific reference genome using the consensus utility within Bcftools. To aid in the identification of DVGs with breakpoints near the 5^ʹ^ or 3^ʹ^ end of a segment, we added a 210nt poly-A pad to the 5^ʹ^ and 3^ʹ^ end of each segment in these reference genomes.

To identify DVG reads that span their respective deletion junction sites, we input into ViReMa v0.25 the sequencing reads that did not align in end-to-end mode. We mapped these reads to their run-specific consensus sequence, using a seed length of twenty-five nucleotides, tolerating one mismatch in the seed alignment and not tolerating any mismatches within eight nucleotides of the junction site. To exclude small indels, we removed any DVG reads that support a deletion of less than 20 nucleotides. Duplicate DVG reads were also removed with ViReMa. Bowtie v1.2.2 (Langmead et al., 2009) was used within ViReMa. The relative read support of each identified DVG was calculated as the ratio of the number of reads supporting a given DVG to the number of reads that align to the central nucleotide of the respective genome segment. Due to the size filter in the sequencing protocol described earlier, we do not expect to identify DVGs that are less than 300 nucleotides in length (including sequencing adaptors and multiplex oligonucleotides).

As discussed in Alnaji et al. (2019), identifying the precise DVG breakpoint from sequencing data can be impossible when there are nucleotide repeats on either side of the deletion junction, as is prone to occur (Alnaji et al., 2021). ViReMa includes a ‘DeFuzz’ feature which allows users to force the reported junction site to either the 5^ʹ^ or 3^ʹ^ end of a given read. However, because DVGs may be supported by reads either in the forward or reverse direction, this behavior results in disparate reporting for the same DVG depending on the supporting read direction. Therefore, we slightly modified ViReMa’s underlying AddToDict function to force the reported junction sites to the 5^ʹ^ or 3^ʹ^ end of the reference genome, instead of the supporting read. All DVG junctions reported here have been DeFuzz’d to the 3^ʹ^ end of the reference genome. ViReMa results were parsed in Perl v5.26.2 using the summary scripts included as part (Alnaji et al., 2019). DVG species are identified by the genomic location (1-indexed) of the nucleotides flanking the deleted nucleotides. For example, PB2 100_800 represents a DVG species generated from the PB2 segment in which nucleotide 101 through 799 have been deleted.

### Statistical analyses and visualization

All statistical analyses were conducted in Python. Statistical tests were conducted using Scipy v1.10.1 (Virtanen, SciPy 1.0 Contributors et al., 2020), and regression modeling was performed using statsmodels v0.13.5 (Seabold and Perktold, 2010). All visualization was done in Python using Matplotlib v3.8.0. For each sample, the diversity (*H*) of identified DVG species (*s*) was calculated in Numpy as a function of the relative read support *r* for all DVG species:


(1) $$ H = -\sum_{i=1}^s \left( \frac{r_i}{\sum_{j=1}^sr_j} \right) \left( \text{ln}(\frac{r_i}{\sum_{j=1}^sr_j}) \right). $$

Evenness was then calculated as:


(2) $$ J = \frac{H}{\text{ln}(s) }. $$

*H* and *J* were calculated only among samples with at least one DVG. Samples with only a single DVG were assigned *H* = 0 and *J* = 1.

The presence of premature stop codons in all observed DVG species within each sample was identified by first generating the sequence of a genome segment with a given DVG deletion (using the run-specific consensus sequence) and translating said sequence to a string of amino acids based on all open reading frames on a given sequence. A DVG was considered as giving rise to a premature stop codon if at least one premature stop codon was inferred across all possible reading frames in a given segment. For each sample, the total relative read support of premature stop codon DVGs in each considered segment was tabulated by summing the relative read support of all DVGs on a given segment that give rise to a premature stop.

A null distribution for the expected relative read support of premature stop codon DVGs was generated by randomizing the breakpoints for each observed DVG. This was done by drawing a random 5^ʹ^ and 3^ʹ^ breakpoint, with replacement, from the observed breakpoints on each reference segment. To avoid recapitulating preferences for specific breakpoints in the empirical data, a noise term, drawn uniformly from [−10, 10], was added to each breakpoint. Rejection sampling was used to ensure that at least 500 nucleotides were deleted in each simulated DVG. This process was repeated 1,000 times. These randomized DVG breakpoints were then applied to each run-specific consensus genome to identify premature stop codons and calculate the relative read support of DVGs with a premature stop codon in a similar manner as above.

To examine the extent to which DVG species had in-frame versus out-of-frame nucleotide deletions, the modulus (‘mod’) of a given DVG was calculated using the $\%$ operator in Python. For example, the DVG PB2 100_800 in which Nucleotide 101 through 799 have been deleted has a mod of $(800-100-1)\%3 = 0$. The proportion of relative DVG reads on each segment was calculated by drawing 10,000 bootstrap replicates of all DVGs observed on that segment, weighted by their relative read support, and calculating the proportion with mod = 0, 1, or 2. The median, 2.5th, and 97.5th percentile of the proportion belonging to each mod category were calculated in Numpy.

The length of the nucleotide repeat flanking a given DVG junction site was calculated based on the run-specific consensus sequence in which a given DVG was observed.

### Transmission analyses

We used the same household pairings as reported in McCrone et al., 2018. Epidemiologically linked pairs (‘transmission pairs’) were identified as pairs of individuals from the same household who were infected with influenza viruses more similar than 95 per cent of unlinked pairs, as measured by the L1-norm. When the direction of transmission was uncertain (*N* = 6 pairs), the couple was considered only once when tabulating the number of shared DVGs, but we considered the relative support of shared and unshared DVGs from both individuals. When multiple samples were available from the same host (regardless of whether donor or recipient, *N* = 25 individuals across 26 transmission pairs), we used the set of DVGs present in all samples for a given host, taking the maximum relative read support. Pairs of epidemiologically unlinked samples (“unlinked pairs”) were generated by randomly assigning pairs of samples within each season, excluding samples from the same household.

### Data and code availability

All raw sequencing data are available from the NCBI SRA BioProject PRJNA412631 (McCrone et al., 2018). Additional metadata are available as part of McCrone et al., 2018 and the associated repository at https://github.com/elifesciences-publications/Host_level_IAV_evolution. All additional analysis and visualization code is available at https://zenodo.org/doi/10.5281/zenodo.11121677.

## Results

### A limited number of DVGs are observed in plasmid controls

To determine the number of spurious DVGs introduced by the sequencing protocol, we first queried the reads generated from the plasmid controls for each sequencing run for DVGs. This analysis acts as a negative control as these DVG reads are generated from clonal plasmids containing full-length viral genome segments and thus should not contain any DVGs. While we observed many (median [sd]: 53.50 [62.66]) unique DVG species in each plasmid control, encouragingly these DVG species were almost exclusively present at very low read support (Figure S1, $1.14\mathrm{e}{-3}$ [sd $7.50\mathrm{e}{-4}$]). The vast majority (>99.67 percent) of DVGs identified in plasmid controls are supported by <0.005 relative reads. Consequently, we required DVGs to be supported by a minimum of 0.005 relative reads for all downstream analyses to remove spurious DVGs introduced by the sequencing protocol. Furthermore, we remove any DVGs identified in the respective plasmid control for a given sequencing run. Notably, as plasmid controls do not have to be reverse transcribed, this analysis cannot control for spurious DVGs present in clinical samples that were generated when converting viral RNA to cDNA.

### DVGs are observed readily in clinical samples

**Figure 1.**
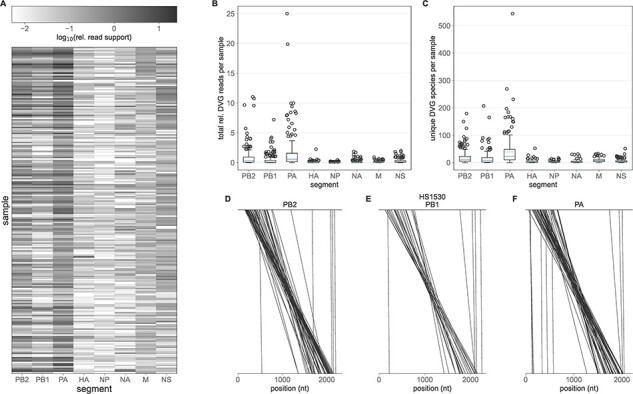
DVGs identified in clinical samples. (A) Heatmap showing the prevalence of DVGs in each sample (rows), by gene segment (columns). DVG prevalence is quantified by the total relative DVG read support across a given segment. (B) Total relative DVG reads per sample per segment. (C) Number of unique DVG species per sample per segment. In (B) and (C), solid lines in the boxplots show the median value for each segment, dotted lines show the mean, and box extends to the limits of the IQR, and whiskers extend to 1.5 IQR below and above the first and third quartiles, respectively. Outliers are shown as dots beyond the range of the whiskers. (D–F) Breakpoints of all PB2 (D), PB1 (E), and PA (F) DVG species identified in representative sample HS1530. Each line connects the last undeleted base on the 5^ʹ^ end of the DVG and the first undeleted base on the 3^ʹ^ end of the DVG.

In contrast to the plasmid control earlier, DVGs are observed readily in the clinical samples (Figure 1A). We observe at least one DVG species above the relative read threshold of 0.005 in all 217 of the clinical samples. Lower viral titer samples tend to harbor more relative DVG reads, potentially reflecting increased technical noise in low titer samples (Figure S2, Table S1). However, this association is only statistically significant at the *α* = 0.05 level in the NP, M, and NS segments (*P*-values <0.001, <0.001, and 0.033, respectively) and the estimated effect sizes are relatively small (e.g. 0.0158 decrease in relative read support per unit decrease in log_10_ genomes/*µ*l in the NP segment). We, therefore, do not expect our DVG abundance estimates to be significant biased by sample titer. Most DVG species are very rare, present in a mean of 1.59 samples, with 47.85 per cent observed in only a single sample (Figure S3A). The most common DVG, NS 316_545, was present in 125 out of 217 samples. Analysis of the unfiltered DVG data revealed that this DVG was observed in an additional eighty-one samples at a relative read support ≥0.005 but was removed by our filtering protocol as it was also observed in the sequence data for the corresponding plasmid control. A BlastN analysis of a representative supporting read and the inferred NS segment with this DVG overwhelming align to influenza A virus segments (Tables S2–S4), indicating that it is not the result of sequencing reads generated from contaminant genetic material. Notably, the junction sites for NS 316_545 occur at an 11-nucleotide repeat in the NS segment, which is highly abnormal among identified DVG species (Figure S4A). We do not observe a general trend between the repeat length adjacent to DVG junction sites and the number of samples in which a given DVG is observed (Figure S4C).

We observe DVGs most abundantly on the PB2, PB1, and PA influenza virus segments (Figure  1A,B, Mann-Whitney U test comparing the relative read support of PB2, PB1, and PA DVGs vs. HA, NP, NA, M, and NS DVGs *P*-value <0.0001). The polymerase segments also harbor about an order of magnitude more unique DVG species compared to the remaining five segments (Figure  1C, Mann–Whitney U test comparing the number of unique PB2, PB1, and PA DVGs vs. HA, NP, NA, M, and NS DVGs in each sample *P*-value <0.0001). Our finding that DVGs are most abundant on the polymerase segments is consistent with previous *in vitro* (Alnaji et al., 2021; Pelz et al., 2021) and *in vivo* (Saira et al., 2013) analyses. Based on this, we limit downstream analyses to DVGs found on PB2, PB1, and PA.

Canonically, the generation of influenza DVGs results in the deletion of the internal coding region for each segment and the conservation of the 5^ʹ^ and 3^ʹ^ termini, which are thought to be needed for virion packaging (Gerber et al., 2014). To assess whether our identified DVGs followed this pattern we mapped the deletion junction sites onto the reference genome (Figure  1D,E,F, Figure S5). This analysis reveals that the majority of observed DVG species do indeed result in the deletion of the internal portion of each segment, providing confidence that our analysis pipeline is accurately identifying defective genomes, that is, those which are incapable of establishing productive infection in the absence of wild-type virus. Nearly all observed DVG species maintain the 5’ and 3’ termini. However, it is important to note that these data were generated by the amplification with primers that bind to these portions of each segment (McCrone et al., 2018; Zhou et al., 2009). As such, identified DVG species may represent only a subset of the DVG population present in a given sample. Our analysis also identified a small number of polymerase DVGs harboring relatively small deletions. A comprehensive analysis of the number of deleted nucleotides in each of the observed polymerase DVGs reveals a highly bimodal pattern (Figure S6). To ensure that our analyses were based solely on truly defective genomes and not those harboring small indels with minimal fitness effects, we implemented an additional empirical filtering step to remove any DVG species with fewer than 500 deleted nucleotides. Among polymerase DVGs with at least 500 deleted nucleotides, we observe a preference for DVG junction locations to occur between locations in the genome with nucleotide repeats of length 1 to 7, as compared to a null distribution of simulated DVGs (Figure S4B,D).

### DVG populations are dynamic

**Figure 2.**
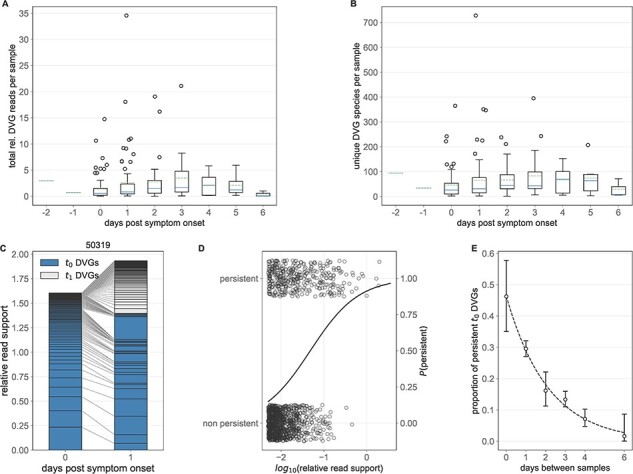
Polymerase DVG dynamics over the course of infection. (A) Total relative polymerase DVG reads per sample as a function of the number of days post symptom onset that a given sample was taken. (B) Number of unique polymerase DVGs per sample as a function of the number of days post symptom onset that a given sample was taken. In (A) and (B), solid lines in the boxplots show the median value for each segment, dotted lines show the mean, box extends to the limits of the IQR, and whiskers extend to 1.5 IQR below and above the first and third quartiles, respectively. Outliers are shown as dots beyond the range of the whiskers. (C) Longitudinal DVG dynamics in Representative Individual 50319. Lines connect the relative read support of a given polymerase DVG in each of the two samples taken. DVGs first observed at the first time point (*t*_0_ DVGs) are shaded and those observed at the second, but not the first, time point (*t*_1_ DVGs) are unshaded. (D) log_10_ relative read support of *t*_0_ DVGs which do and do not persist at time *t*_1_ for longitudinal samples from the same subject 1 day apart. Solid line represents the estimated probability of a DVG with a given log_10_ relative read support persisting from a logistic regression. (E) Proportion of all DVGs observed in a given *t*_0_ sample that are also observed in the corresponding *t*_1_ sample as a function of the number of days between when those samples were taken. Whiskers extend to the exact binomial confidence intervals for a given proportion.

Our primary goal in this study was to assess how DVG populations change over time during the course of single infections and during transmission between hosts. To assess the former of these two goals, we first analyzed both the total relative polymerase DVG read support as well as the number of unique polymerase DVG species on a per-sample basis as a function of the days post-symptom onset, which is used in the absence of data on time since infection (Figure 2A,B). Between 0 and 3 days post symptom onset, we do observe an increase in the quantity of relative DVG reads (mean [sd] 1.60 [2.64] vs. 3.45 [4.56], *P*-value = 0.003) and the number of DVG species (45.95 [61.89] vs. 83.0 [90.25], *P*-value = 0.01) per sample. By Day 6, we observe a precipitous drop in the quantity of DVG reads (0.39 [0.45]) and number of DVG species (28.67 [30.65]), likely due to the decreased viral population size toward the end of infection. This trend is qualitatively similar to observed numbers of iSNVs identified in these samples, which appear, by eye, to increase between Day 0 and Day 4 post symptom onset and decrease by Day 6 (McCrone et al., 2018).

To examine patterns of DVG generation and persistence within individuals, we examined the DVG populations identified in the subset of individuals with longitudinal data (*N* = 49 individuals, each with 2 sampling times). In general, DVG populations are highly dynamic over time, from one collection time point (*t*_0_) to the next (*t*_1_) (Figure 2C and Figure S7) and many DVG species do not persist across time points. These observed DVG dynamics likely stem from a combination of real changes in the underlying population and technical noise induced by sampling, reverse transcription of viral RNA, and amplification of cDNA (Boussier et al., 2020). Nevertheless, the extinction of many *t*_0_ DVG species and the appearance of novel DVG species at *t*_1_ implies that there is continuous *de novo* DVG generation and loss during infections.

**Figure 3.**
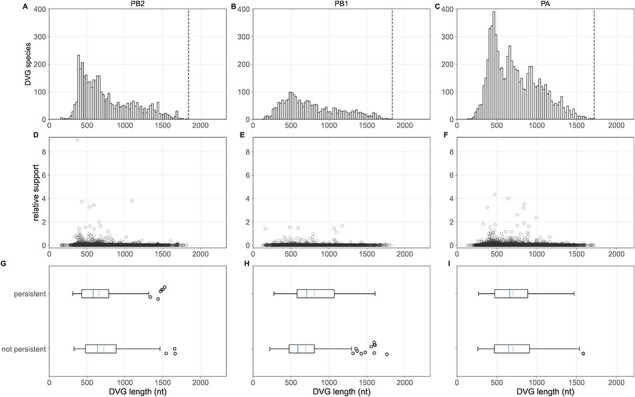
Length patterns of observed polymerase DVGs. Top row: Length of all observed DVG species in the PB2 (A), PB1, (B), and PA (C) segments, binned in 25 nucleotide bins. Vertical dotted line shows our threshold for calling a ‘defective’ genome. Middle row: Relative read support of DVG species in the PB2 (A), PB1 (B), and PA (C) segments plotted as a function of their length. Bottom row: PB2 (G), PB1 (H), and PA (I) DVGs observed at the first time point in the 24 individuals with available sequence data on consecutive days (*N* = 24) stratified by whether they are observed at the second time point (‘persistent’) or not (‘not persistent’) plotted as a function of their length. Solid lines in the boxplots show the median value for each segment, dotted lines show the mean, and box extends to the limits of the IQR, and whiskers extend to 1.5 IQR below and above the first and third quartiles, respectively.

We first hypothesized that the probability that a DVG persists across longitudinal time points is dependent on the frequency of that DVG at the first time point. Using logistic regression, we tested whether the log-odds that a DVG identified in the first sample of a pair was still present in the second sample of the pair depended on the log_10_ relative read support of the DVG identified in the first sample. Among samples taken 1 day apart (*N* = 24), we find that read support is significantly associated with the log odds of DVG persistence (*P*-value < 0.001, Figure 3D, Table S5) such that a 1 log-unit increase in the relative read support is associated with an odds ratio of persistence of 1.78. Specifically, between a log_10_ relative read support of –2 and 0, the probability of DVG persistence increases from 0.23 to 0.91.

There are a range of time intervals between the two samples (from 0 to 6 days) available for individuals with longitudinal data. These longitudinal data include self- or parent-collected nasal swabs at illness onset and combined nasal and throat swabs at a later visit to the research clinic (see McCrone et al., 2018). Thus, we are able to correctly specify the temporal ordering of samples even among those taken on the same day. To evaluate the impact of the time between samples on the probability of DVG persistence, we examined how quickly DVGs that were identified in one sample were no longer observed in a later sample. To this end, we identified the unique DVG species present in the earliest sample (*t*_0_) from each individual with two samples in this dataset and calculated the proportion of these which were present in their later (*t*_1_) sample. By grouping these proportions based on the time between samples, we find a significant dependence on the number of days which have elapsed between *t*_0_ and *t*_1_ (*χ*^2^ test of independence *P*-value < 0.001, Figure 2E). Specifically, the further apart in time that the two samples from a host were taken, the fewer *t*_0_ DVGs persisted in the *t*_1_ sample. Of DVGs observed at *t*_0_, 28.93 percent are present a day later and only 2.75 percent persist 6 days later. These result are further supported by a multivariate logistic regression model incorporating both relative read support and time between samples as a categorical variable (Figure S8 and Table S6). In this model, both relative read support and time between samples are significant predictors of the probability of DVG persistence (all *P*-values <0.001).

### No evidence for selection acting on DVG species *in vivo*

Previous *in vitro* work has observed a preference for the replication of shorter DVG species that is shaped by length-based selection during virion packaging (Alnaji et al., 2021; Pelz et al., 2021; Mendes and Russell, 2021). To evaluate whether we observe any length-based selection in *in vivo* DVG populations, we first determined the length of all unique DVG species in each subject (Figure 3A–C). We observe a clear preference for DVG species that are shorter in all three polymerase segments, although our data are truncated by the size filtration step in the laboratory methods used to generate these data (McCrone et al., 2018). Among DVGs that are observed, shorter ones tend to be observed at higher relative read support (Kruskal–Wallis H-test *p*-values <0.001, Figure S9A–C). While these patterns may reflect a real preference for shorter DVG species, they may also be biased by preferential PCR amplification of shorter genetic segments (Shagin et al., 1999; Boussier et al., 2020).

Based on this cross-sectional analysis alone, we are unable to disentangle whether the observed preference for shorter DVG species is due to preferential within-host generation or selection following generation. However, we can use longitudinal data to address this question. To do so, we identified all DVGs present in the first-time sample of the 24 individuals with paired sequence data sampled 1 day apart and identified whether they were observed in the second sample (‘persistent’) or not (‘not persistent’). If shorter DVGs were under positive selection, we would expect them to be more likely to persist across time points. This analysis, however, reveals no consistent preference for the persistence of shorter DVGs. In PB2, persistent DVGs tend to be slightly shorter (mean [sd] 660.30 [297.66] vs. 721.07 [304.45], *P*-value = 0.02), whereas they are slightly longer in PB1 (806.89 [328.77] vs. 693.79 [333.20], *P*-value = 0.01) and indistinguishable in PA (700.32 [272.39] vs. 700.17 [280.51], *P*-value = 0.86). As above, we again fit a multivariate model predicting DVG persistence as a function of time between sampling, log_10_ relative read support at *t*_0_, and DVG length. In these models, DVG length was not a significant predictor of DVG persistence (Figure S9D, Table S7, and Table S8), with the exception of DVGs between 750 and 1250 nucleotides in length when modeled as a categorical variable (*P*-value=0.02). Given this combined set of results, we conclude that evidence for length-based DVG selection in these data is limited.

**Figure 4.**
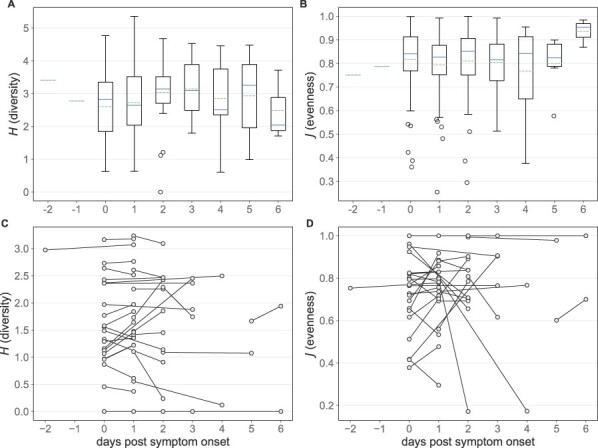
Longitudinal trends in polymerase DVG diversity and evenness. (A) Diversity of DVG populations among all samples stratified by time since symtpom onset. (B) Evenness of DVG populations among all samples stratified by time since symptom onset, excluding samples in which only one DVG was identified. In (A) and (B), solid lines in the boxplots show the median value for each segment, dotted lines show the mean, and box extends to the limits of the IQR, and whiskers extend to 1.5 IQR below and above the first and third quartiles, respectively. Outliers are shown as dots beyond the range of the whiskers. (C) Diversity at both sampling times of the subset of DVGs present in the first sample among individuals with longitudinal sampling. (D) Evenness at both sampling times of the subset of DVGs present in the first sample among individuals with longitudinal sampling, excluding individuals with only one DVG at the first sampling time.

To evaluate whether there is any evidence of selection acting on other DVG features, we next evaluated trends in DVG diversity and evenness over the course of infection. As noted above, DVG populations within hosts are highly variable. Were there to be selection acting on specific DVG species, we might expect to see a reduction in DVG diversity and evenness throughout the course of infection. Across the individuals in this dataset, however, we see no such trend (Figure  4A,B, Kruskal–Wallis H-test *P*-value = 0.32, 0.43, respectively).

This cross-sectional analysis is unable to differentiate between the effects of selection reducing DVG diversity and evenness and continual generation of new DVGs throughout an infection. To disentangle these effects, we again turn to individuals with paired longitudinal sequence data. Specifically, we focus on the subset of DVGs that are present at the first time point and estimate the diversity and evenness of those DVGs at both sequenced time points. Among this population of DVGs, we observe no clear trend toward a decrease in diversity or evenness between time points, regardless of when in the course of infection samples were taken (Figure  4C,D). This pattern is consistent with lack of evidence for selection acting on the DVG populations present at *t*_0_.

Finally, *in vitro* work has suggested that some DVG species may be translated to peptides and small proteins and potentially serve a functional role during infection (Akkina et al., 1984). To determine whether there was any signal for this in these data, we first evaluated whether there was any preference for DVGs that delete a factor of three nucleotides, thereby preserving the original reading frame. When DVG reads are stratified by the number of deleted nucleotides (*D*) mod ($\%$) 3, we do not observe a consistent preference for DVGs where $D \% 3 = 0$ (Figure S10A–C). Furthermore, individual DVGs with $D \% 3 = 0$ are not observed at higher relative read support than those with $D \% 3 = 1 \text{ or } 2$ (Mann–Whitney U test comparing relative read support of DVGs with $D\%3 = 0$ to those with $D\%3 = 1 \text{ or } 2$ on PB2, PB1, and PA segment *P*-value = 0.66, 0.09, 0.54, respectively, Figure S10D–F). We further evaluated whether there was any selection against DVGs that result in a premature stop codon, which may be expected if there is selection acting on the translated product of DVGs. In general, however, the total relative read support of DVGs with premature stop codons in each sample closely follows what would be expected from a null distribution of randomly generated DVGs (Figure S11A–C). Furthermore, DVG read support does not seem to differ between observed DVGS that do versus do not have premature stop codons (Mann–Whitney U test comparing relative read support of DVGs with and without premature stop codons on PB2, PB1, and PA segment *P*-value = 0.40, 0.06, and 0.74, respectively, Figure S11D–F). Thus, we do not see any positive evidence for selection acting against DVGs based on the presence/absence of early stop codons.

Taken together, our analyses have not revealed evidence for selection acting on influenza A virus DVGs generated during natural human infection. Within-host DVG persistence appears to be governed primarily by the time between samples and the the abundance of the DVG species within a sample. These findings are consistent with a model in which within-host viral populations (including DVG subpopulations) during acute infections are dominated by genetic drift as opposed to selection.

### DVGs support the existence of a tight transmission bottleneck

Viral evolution is shaped not just by the dynamics which operate within hosts but also by those which operate between hosts, including at the stage of transmission. Previous analyses based on viral genetic variation have revealed that the transmission bottleneck of influenza A is quite small, on the order of one to two virions (McCrone et al., 2018; Shi et al., 2024). This results in a significant loss of genetic diversity during transmission such that nearly all genetic variation observed within a host is likely to have been generated *de novo* following transmission.

**Figure 5.**
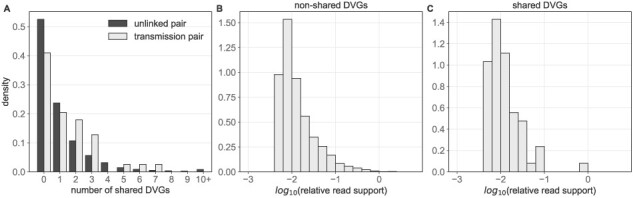
Polymerase DVGs shared between pairs of individuals. (A) Histogram showing the number of DVGs shared between epidemiologically unlinked pairs (dark shading) and between transmission pairs (light shading). X-axis has been truncated to 10 for visualization purposes, despite a long tail on the unlinked pair distribution. (B) Relative read support in the source individual of DVGs that are not shared between donor and recipient individuals. (C) Relative read support in the source individual of DVGs that are shared between donor and recipient individuals.

To determine whether genomic diversity supports these conclusions, we evaluated how DVG populations compare between known donor and recipient pairs. To do this, for each transmission pair, we tabulated the number of unique DVG species identified in the donor that were also identified in the recipient (and thus could have been transmitted from donor to recipient). We compared this to a null distribution of epidemiologically unlinked samples to account for the *de novo* generation of identical DVGs within each host. We found that epidemiologically unlinked samples generally share very few polymerase DVGs, on the order of zero to two (mean [sd] = 1.08 [1.99], Figure 5A). This indicates that *de novo* DVG generation on polymerase segments does not result in the repeated emergence of a small number of specific DVGs as expected based on Figure S3. Among transmission pairs, we similarly found that generally very few DVGs are shared between individuals (1.41 [1.73]). The number of DVG species shared between random pairs and household pairs was not found to be significantly different at an *α* = 0.05 level (Mann–Whitney U test *P*-value = 0.07). With a hard cut-off for significance, this indicates that transmission pairs are not statistically more likely to share more DVGs than unlinked pairs. However, we can also interpret our findings more cautiously: transmission pairs might share more DVGs than unlinked pairs; however, if they do, the excess number they share is small, indicating that very few DVG species, if any, transmit between donor and recipients. This finding is consistent with a small transmission bottleneck that has been inferred using patterns of viral genetic diversity.

To further assess the possibility of DVG transmission between donors and recipients, we analyzed in more detail the 55 DVG species that were shared across the 23 transmission pairs. If these DVGs were shared due to transmission as opposed to *de novo* generation, we would expect them to be disproportionately present at higher relative read support in the donor host relative to DVGs that were not transmitted. However, we observe that the relative read support of shared DVGs (mean [sd] 0.030 [0.11]) is comparable to that of non-shared DVGs (0.031 [0.20]) We found no significant difference between the relative read support in the donor host of shared and non-shared DVGs (Figure 4B,C, Mann–Whitney U *P*-value = 0.78).

These results indicate that DVGs are unlikely to be transmitted from donors to recipients and thereby support the conclusion from previous studies based on viral genetic variation that transmission bottleneck sizes are very small.

## Discussion

Understanding how influenza viral populations evolve within and between hosts is key to understanding how viral evolution proceeds on the host population scale. This is relevant, for example, in learning how new antigenic variants arise and ultimately sweep the host population. Previous work has used viral genetic diversity, or diversity in the form of single nucleotide variants, to better understand the evolutionary forces acting on virus populations within hosts. These analyses have not found evidence of positive selection acting efficiently within acutely infected hosts, e.g. known antigenic escape mutants do not appear to be enriched in vaccinated individuals (Debbink et al., 2017; McCrone et al., 2018). Purifying selection appears to occur to some extent. Genetic drift is thought to be strong in these populations, underscoring the role that stochasticity plays in shaping viral evolution within hosts. Furthermore, the size of the transmission bottleneck has been estimated to be on the order of one to two virions (McCrone et al., 2018; Shi et al., 2024). This stringent bottleneck introduces an additional source of genetic drift at the point of transmission. However, the resolution of these studies is inherently limited by the low levels of genetic diversity that exist within acute human infections of influenza (Debbink et al., 2017; McCrone et al., 2018).

Analyses based solely on iSNVs do not consider the *genomic* diversity that is generated during infection in the form of DVGs. These genomes feature large internal deletions in at least one segment and are therefore incapable of replicating without coinfection of a cell already harboring a wild type virus. Due to this reliance on coinfection and the spatial structure of within-host infections that may retain linkage between specific wild-type genotypes and their corresponding DVG species, we expect the ecological and evolutionary dynamics of DVGs to mirror those of the wild-type viral population.

At present, little is known about the *in vivo* dynamics of influenza DVGs. The vast majority of our understanding of influenza DVGs come from *in vitro* studies (Wasik et al., 2018; Pelz et al., 2021; Alnaji et al., 2021), and existing *in vivo* studies offer only a cross-sectional view of DVGs within a host population (Saira et al., 2013). How DVG populations change over time, and what those dynamics tell us about the forces shaping the entire collection of influenza viral particles within and between hosts in natural human infections therefore remained an open question. Here, we attempted to address this knowledge gap by identifying DVGs from deep sequencing data collected as part of a longitudinal influenza household cohort study (McCrone et al., 2018). We identified at least some quality-filtered DVGs in all samples in the dataset, primarily on the PB2, PB1, and PA segments. While most DVGs were shared between very few samples, a single DVG, NS 316_545, was observed in 125 out of 217 samples. This DVG occurred in an additional 81 samples but was removed by our filtering as it was also observed in the respective plasmid control. We do not expect this DVG to be due to contamination due to our stringent read filtering and the observation that NS 316_545 supporting reads are assigned to be of influenza origin by BlastN. We therefore suspect that its presence may be driven by spurious generation during our sequencing protocol. Alternatively, the commonality of this DVG could be due to intrasegment recombination, which has been reported to occur in other orthomyxovirus species (Cárdenas et al., 2020) but is thought to be rare in influenza viruses (Boni et al., 2010). This DVG, however, does occur between an 11-nucleotide repeat which may promote recombination (Urbanowicz et al., 2005).

Following their identification, we assessed changes in DVG populations over the course of infection and across transmission pairs. We observed a slight increase in the number of DVG species and the abundance of DVGs through 3 days post symptom onset, with a subsequent decrease at Day 6 post symptom onset. We found evidence for the continual loss and generation of DVGs, with the rate of loss of a DVG species between time points dependent on the DVG’s abundance, as measured by total relative read support.

Furthermore, we did not find positive evidence of selection acting on observed DVG populations. While shorter DVGs do tend to be observed more frequently and at higher relative read support, they are no more likely to persist between time points in single individuals in our longitudinal data. This result is consistent with a potential preference for the generation of shorter DVGs but the absence of positive selection following generation. We observe no decrease in DVG diversity or evenness throughout the course of infection, again leaving us empty-handed in terms of evidence for selection acting directly on DVG populations. Finally, we observe no preference for in-frame DVGs or those that prevent the generation of a premature stop codon. These analyses indicate that in the absence of selection, stochastic processes dominate the dynamics of influenza A virus DVGs during natural human infections.

The strength of genetic drift acting on a given population can be quantified by the effective population size *N*_*E*_. Drift is stronger in populations with small *N*_*E*_ whereas the ability of stochasticity to considerably affect evolutionary dynamics will be minimal in populations with large *N*_*E*_. Quantifying the within-host *N*_*E*_ of influenza A using genetic data is difficult, given the rapidly changing population sizes and noise in iSNV frequency estimates. When it has been attempted, the estimates tend to be on the order of <100 (McCrone et al., 2020). While here we do not attempt to quantify *N*_*E*_, our observations that DVG populations are highly dynamic and change rapidly between time points is consistent with a relatively small *N*_*E*_ as populations with a large *N*_*E*_ would be expected to be more stable.

As discussed earlier, the viral diversity which is present within hosts is also shaped by the process of transmission between hosts. The size of the transmission bottleneck can be quantified to guide our understanding of how this process shapes viral diversity. Here, we compared DVG populations between known transmission pairs. We find that known transmission pairs, on average, share very few DVG species, only marginally more than epidemiologically unlinked pairs. The ones that are shared between donor and recipient pairs are not present at particularly high frequency in the donor, indicating that some of these shared DVGs may be due to *de novo* generation in the recipient host, rather than transmission. These findings underscore the existence of a tight transmission bottleneck of only a small number of viral particles (McCrone et al., 2018; Shi et al., 2024). While our finding that very few, if any, DVGs transmit may appear unsurprising, DVGs can persist in cells *in vitro* for several weeks (Cane et al., 1987), increasing the likelihood that transmitted DVGs would be able to find a wild-type helper virus sometime during the course of infection. Furthermore, it is thought that viruses may transmit not independently, but in collective infectious units of multiple viral particles, which would make it more likely that a DVG and a wild-type virus from the same collective infectious unit find the same host cell (Sanjuán et al., 2017). Our finding that DVG transmission is very rare is consistent with the existence of a very stringent transmission bottleneck and indicates that small transmission bottlenecks may benefit a viral population by purging viral ‘cheaters’ in the form of DVGs (Zwart and Elena, 2015).

There are some important limitations to our analyses. First, as with any *in vivo* deep sequencing–based study, the samples taken from individuals using throat or nasal swabs may not adequately reflect the within-host viral population, particularly when sub-compartmentalization within a host is substantial as has been shown in experimentally infected animal hosts (Amato et al., 2022; Ganti et al., 2022). Additionally, viral genetic material in a given sample was subject to reverse transcription and cDNA amplification, which may result in the generation of spurious DVG species, the loss of species present in the sample, and obfuscation of the population composition (Boussier et al., 2020). In part due to this technical noise, it is difficult to reliably quantify the absolute amount of DVGs within a sample using sequencing data. Here, we have relied on a relative measure of the number of reads spanning a given junction site to the number of reads mapping to the central nucleotide in a given segment as a proxy for the quantity of a DVG species. Without additional laboratory measurements, we feel that this metric represents a suitable attempt to account for varying sequencing depth between samples. Furthermore, our set of identified DVGs will be affected by the amplification and sequencing protocol used to generate these data. Genetic material was amplified based on primers which bind to the terminal regions of the wild-type segments, and there are several size-filtering steps which eliminate smaller DVG segments. However, DVGs that lack these terminal packaging signals would be expected to be inefficiently packaged into virions and would likely be under lethal selection (Gerber et al., 2014; Liang et al., 2005). Sequence-based analysis of *in vitro* DVGs without the size filtration step similarly observed conservation of these packaging signals (Pelz et al., 2021; Alnaji et al., 2019; Saira et al., 2013). We therefore expect the magnitude of bias introduced by the sequencing protocol into our observed distribution of DVG lengths and junction sites to be minimal. Despite these limitations, the fact that the plasmid controls harbor very low relative amounts of DVGs provides confidence that the DVGs reported here represent a subset of true biological DVGs. Alternative viral sequencing approaches, particularly long read sequencing, have the potential to overcome some of these shortcomings and in future studies may provide greater resolution into the evolutionary processes shaping within- and between-host viral populations.

## Supplementary Material

veae042_Supp

## Data Availability

Sequencing data are available at the NLM NCBI SRA under BioProject PRJNA412631. Additional metadata are available at doi:10.7554/eLife.35962.
