# Supplementary material for: Influenza A genomic diversity during human infections underscores the strength of genetic drift and the existence of tight transmission bottlenecks
Source: Virus Evol. 2024 Jun 1;10(1):veae042. doi: 10.1093/ve/veae042 (PMC11179161; doi:10.1093/ve/veae042)
Supplement: veae042_Supp [file veae042_supp.zip › suppl_data/influenza_human_dvg_supplement_combined.pdf]

Supplementary Material: Influenza A genomic diversity during human infections underscores the strength of genetic drift and the existence of tight transmission bottlenecks

Michael A. Martin*, [1,2,3], Nick Berg[3,4,5], Katia Koelle[3,6]

1 Department of Pathology, Johns Hopkins School of Medicine, Baltimore, MD, USA

2 Graduate Program in Population Biology, Ecology, and Evolution, Emory University, Atlanta, GA, USA

3 Department of Biology, Emory University, Atlanta, GA, USA

4 Department of Biochemistry, Brandeis University, Waltham, MA, USA

5 National Institute of Allergy and Infectious Diseases, National Institutes of Health, Bethesda, MD, USA

6 Emory Center of Excellence for Influenza Research and Response (CEIRR), Atlanta GA, USA

* mmart108@jhmi.edu

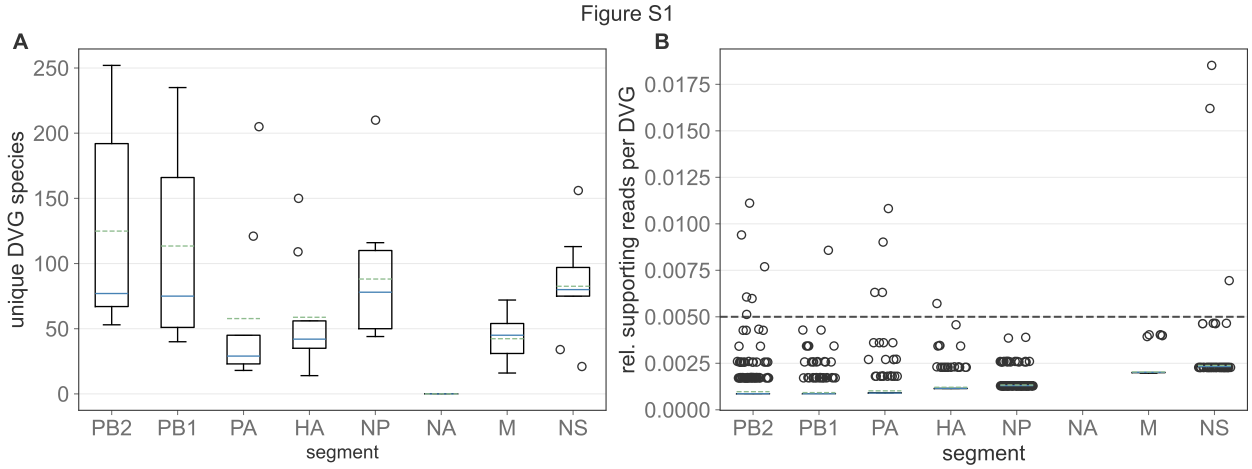

Supplementary Figure 1. DVGs identified in the plasmid controls. Boxes extend to the limits of the inter-quartile range (IQR), and whiskers extend to 1.5 IQR below and above the 1st and 3rd quartile, respectively. Outliers are shown as dots beyond the range of the whiskers. Blue line represents median values and dotted green lines show the mean. (A) Number of DVG species identified per sequenced plasmid per genome segment. (B) Relative DVG read support per sequenced plasmid per genome segment. Dotted horizontal line at 0.005 relative reads represent threshold used for downstream analyses.

Figure S2

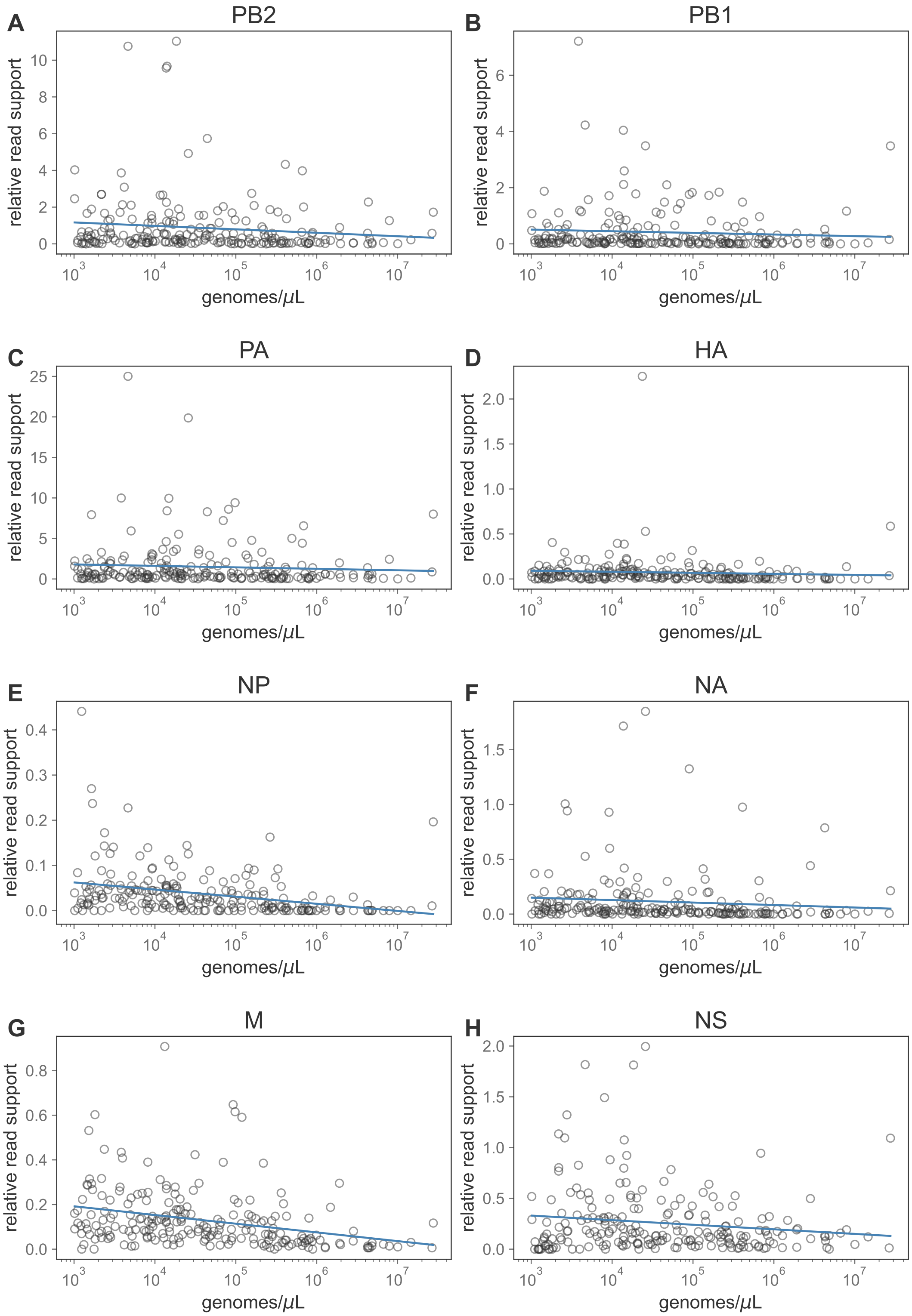

Supplementary Figure 2. Abundance of relative DVG reads as a function of sample titer. Total relative read support of all DVGs on the PB2 (A), PB1 (B), PA (C), HA (D), NP (E), NA (F), M(G), and NS (H) segments as a function of the sample titer of the M-segment for that sample.

Figure S3

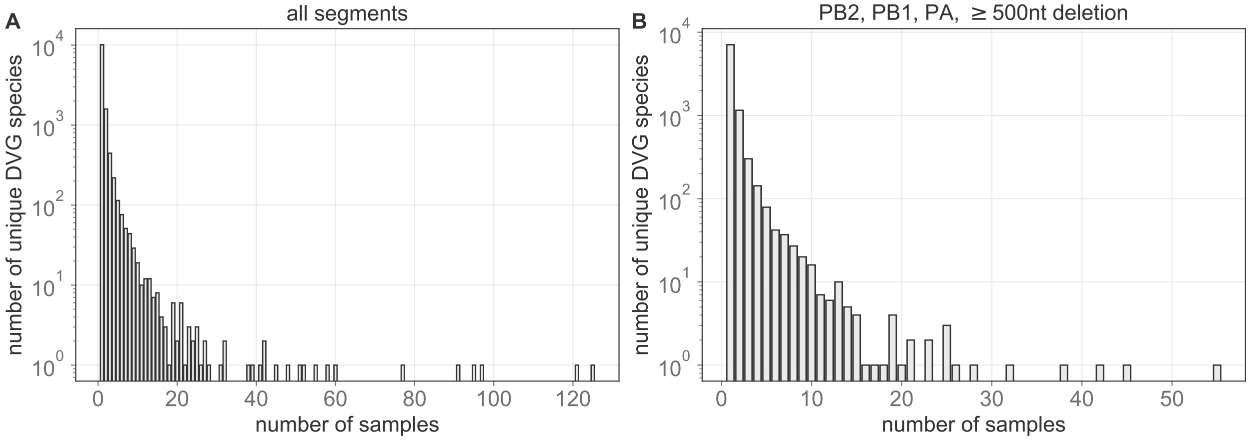

Supplementary Figure 3. Prevalence of unique DVG species. Number of clinical samples in which each unique DVG species are found. (A) DVGs on all segments. (B) Polymerase DVGs with at least 500 nucleotides deleted. Y-axis is shown on the $\log_{10}$ scale.

Figure S4

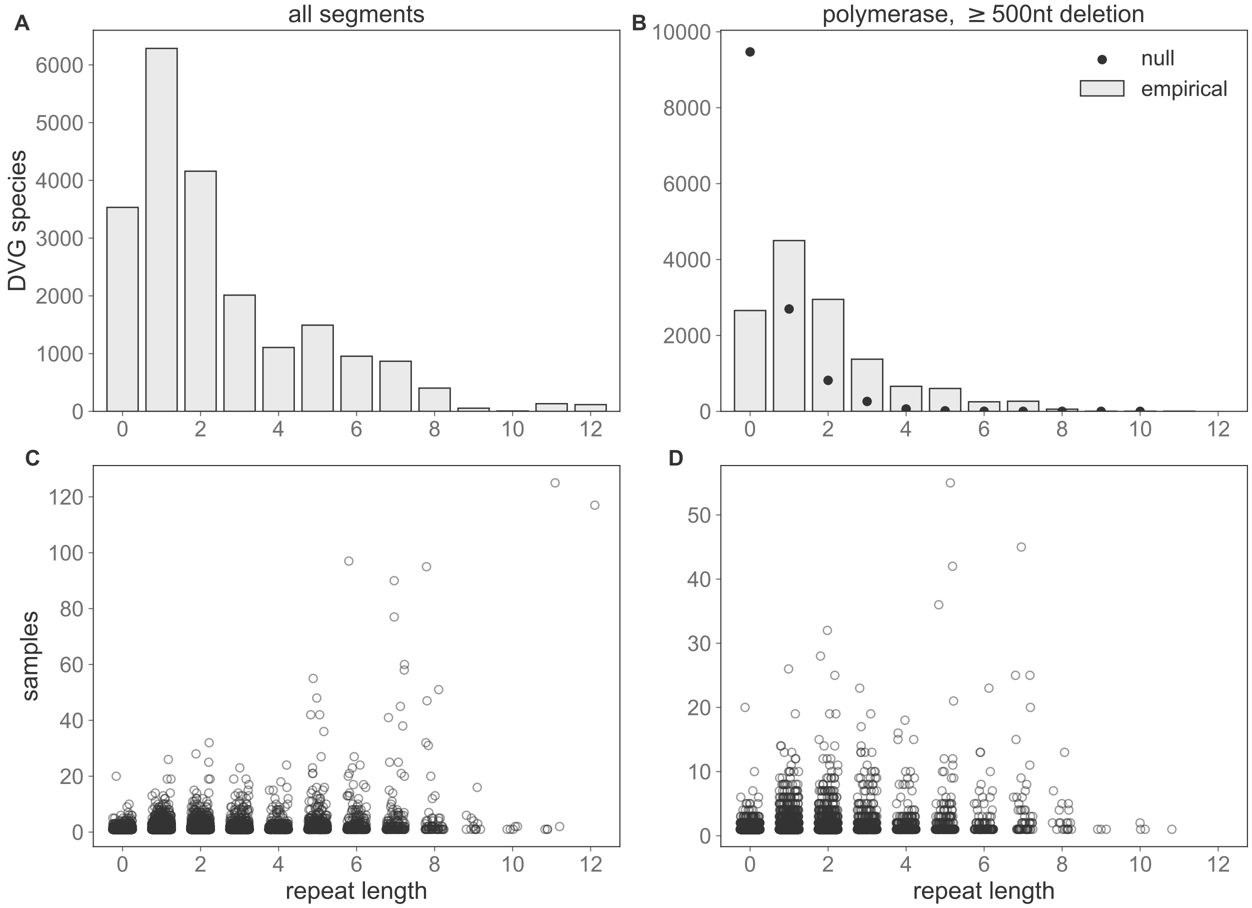

Supplementary Figure 4. Nucleotide repeats at DVG junctions sites. Length of nucleotide repeat observed at the junction site of all DVGs (A) and all polymerase DVGs with at least 500 nucleotides deleted (B). In (B) estimates from the null distribution are shown as black dots. Error bars show the 95th percentile ranges, but do not extend past the size of the points. Number of samples in which each DVG is observed, stratified by repeat length in all DVGS (C) and all polymerase DVGs with at least 500 nucleotides deleted (D).

Figure S5 page 1

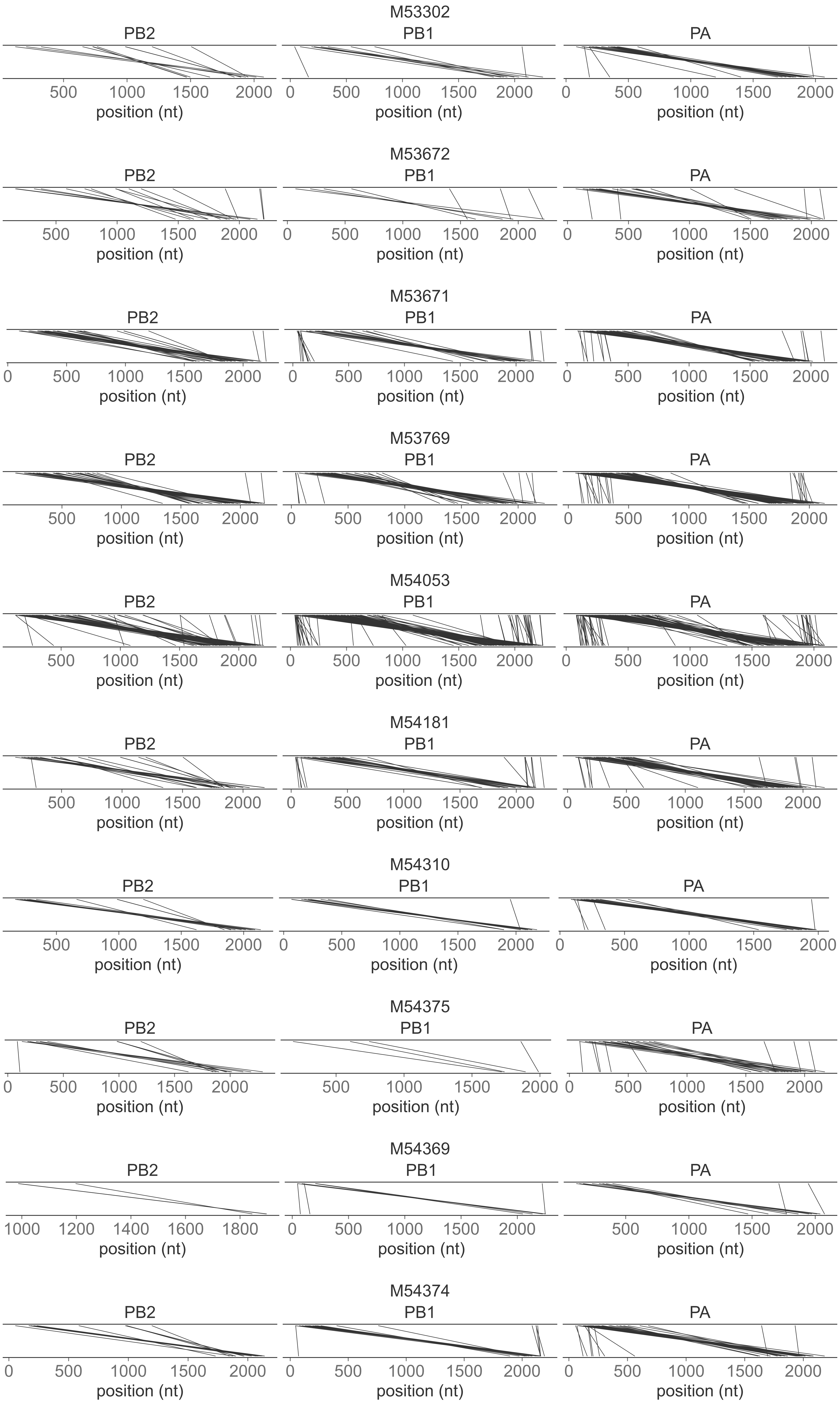

Figure S5 page 2

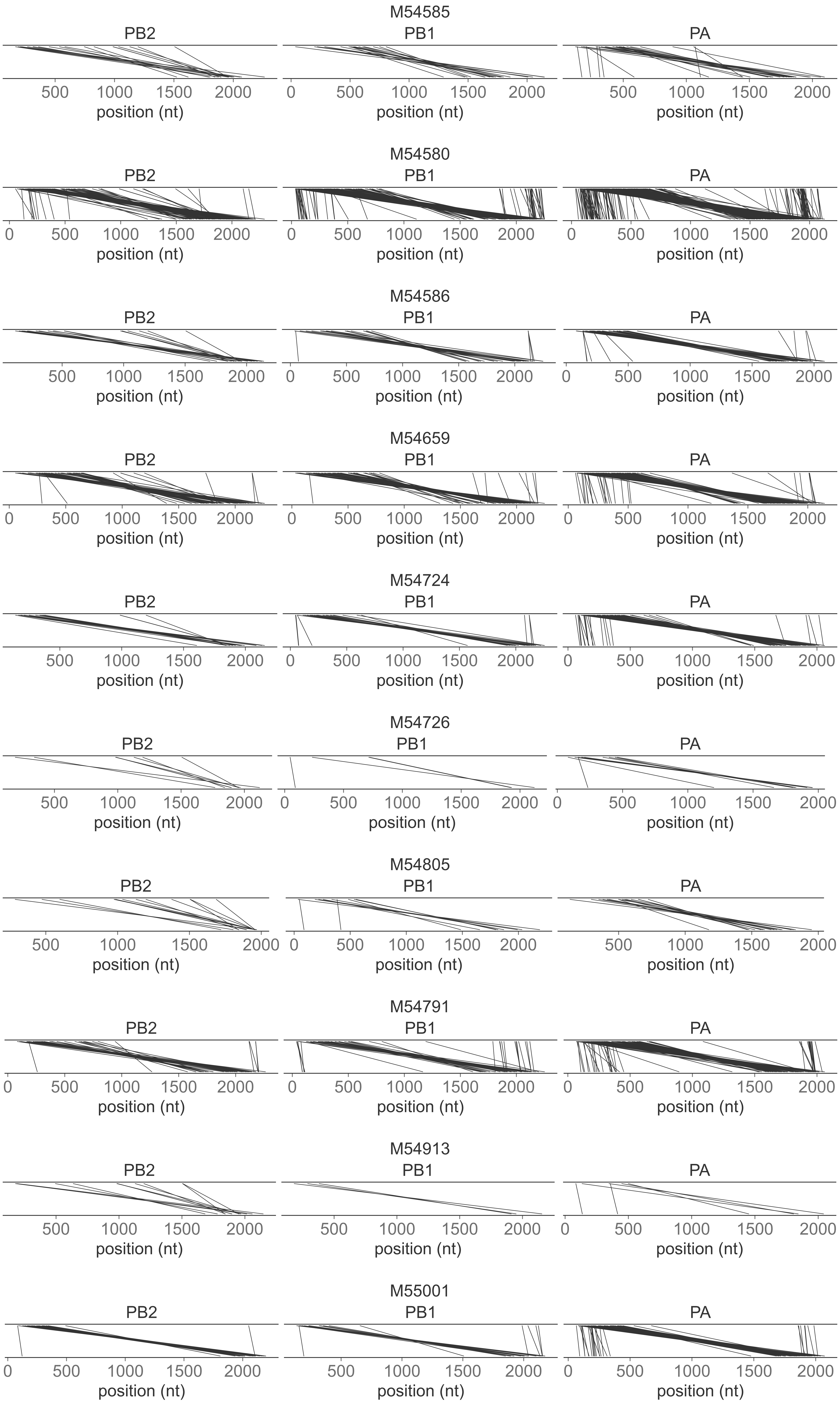

Figure S5 page 3

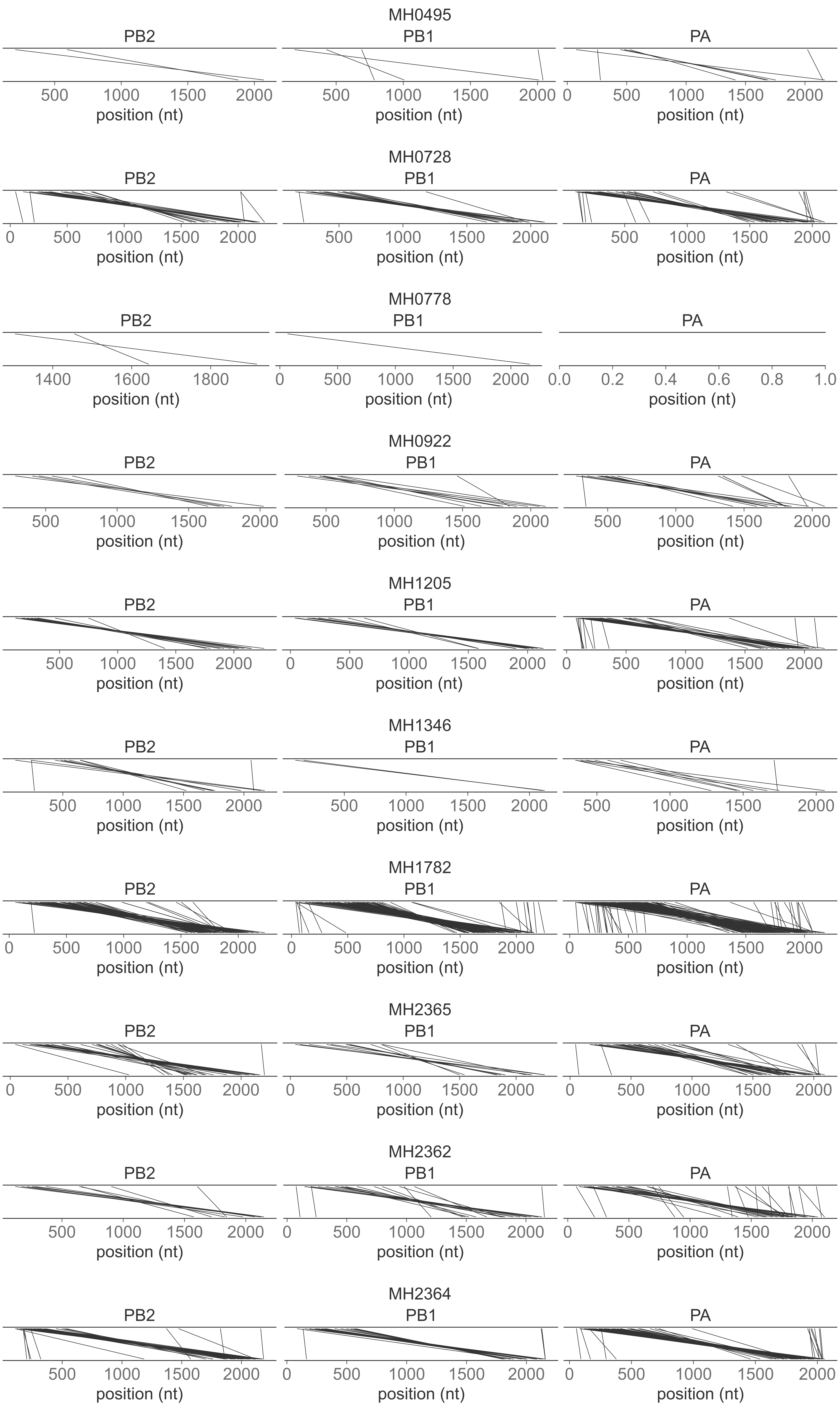

Figure S5 page 4

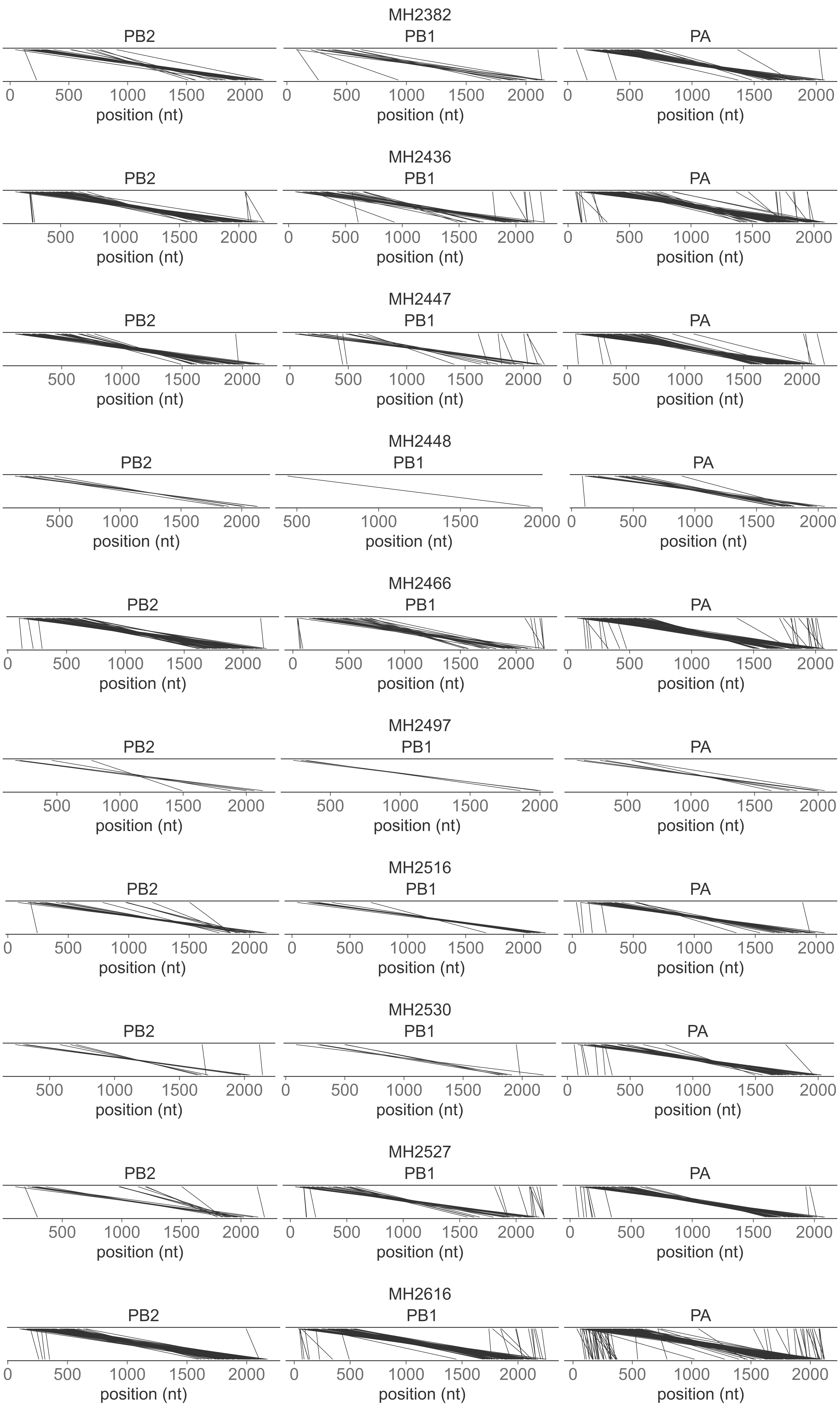

Figure S5 page 5

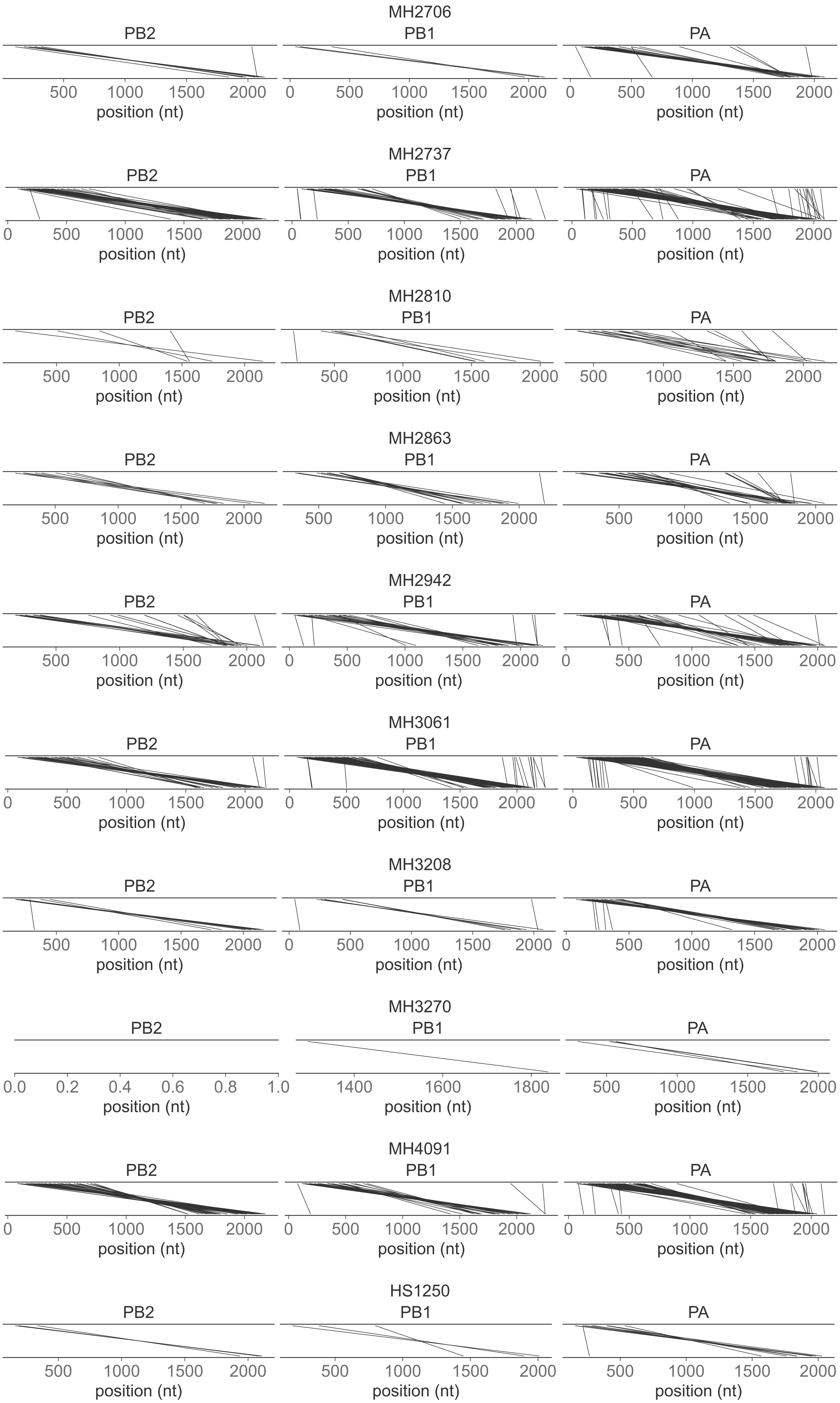

Figure S5 page 6

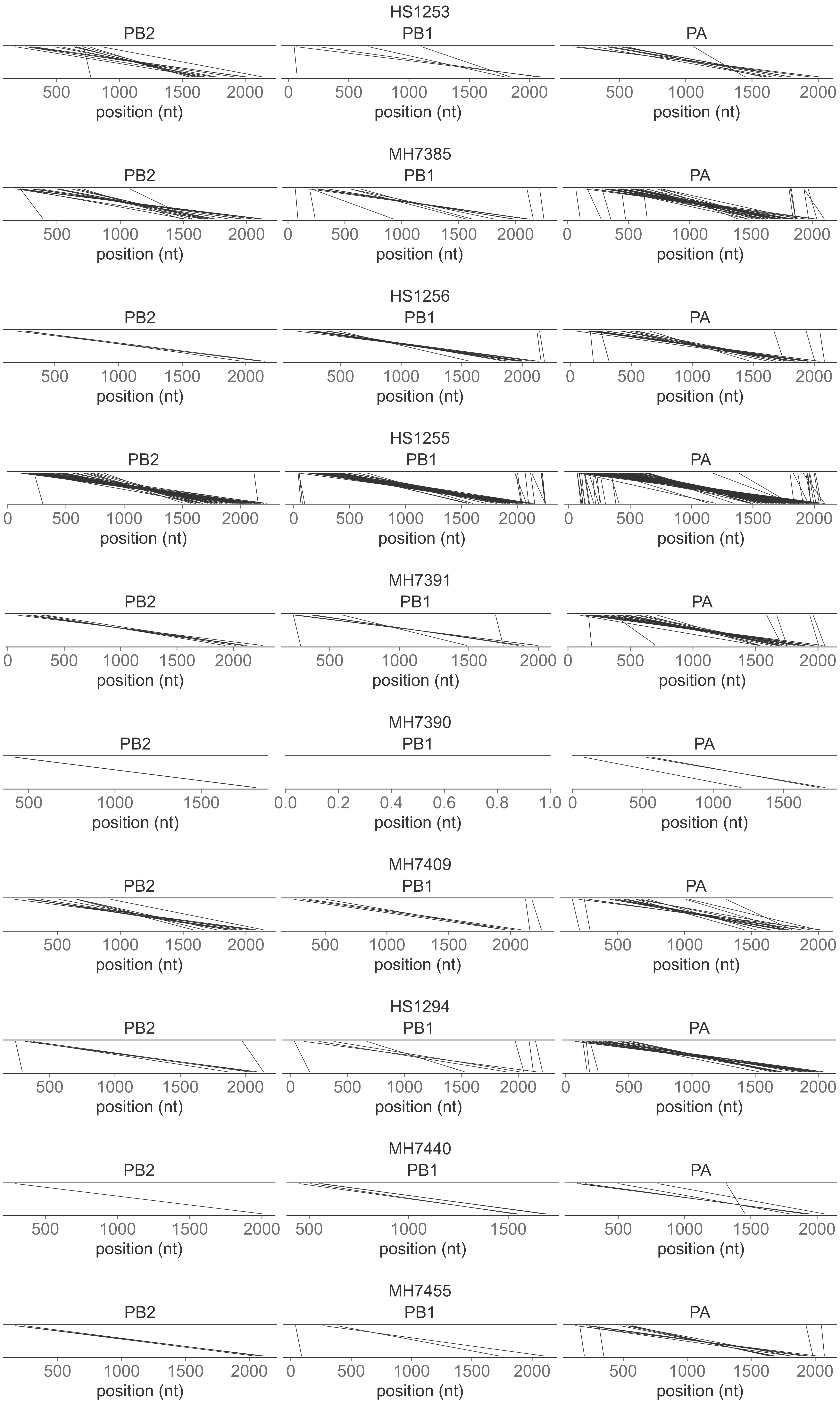

Figure S5 page 7

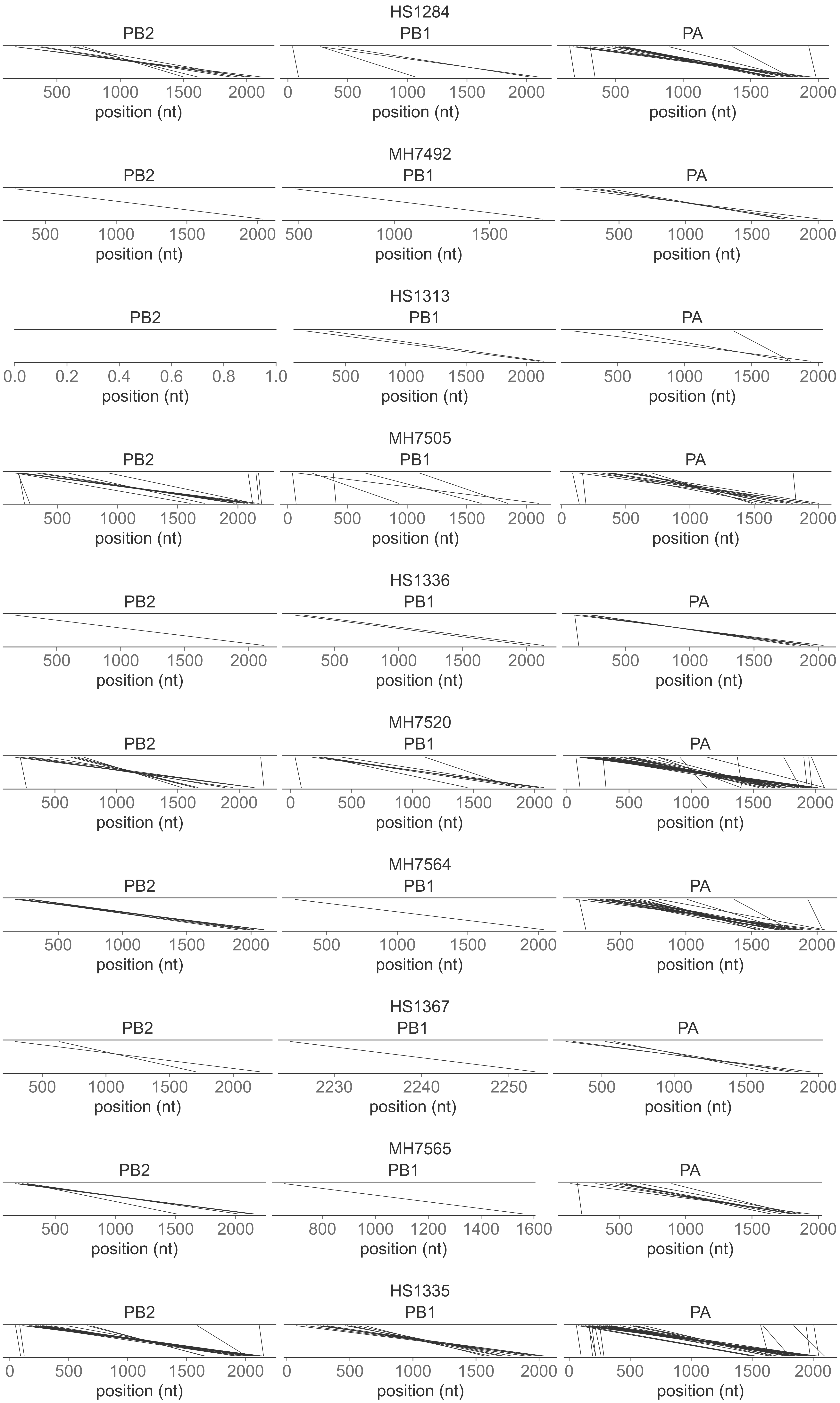

Figure S5 page 8

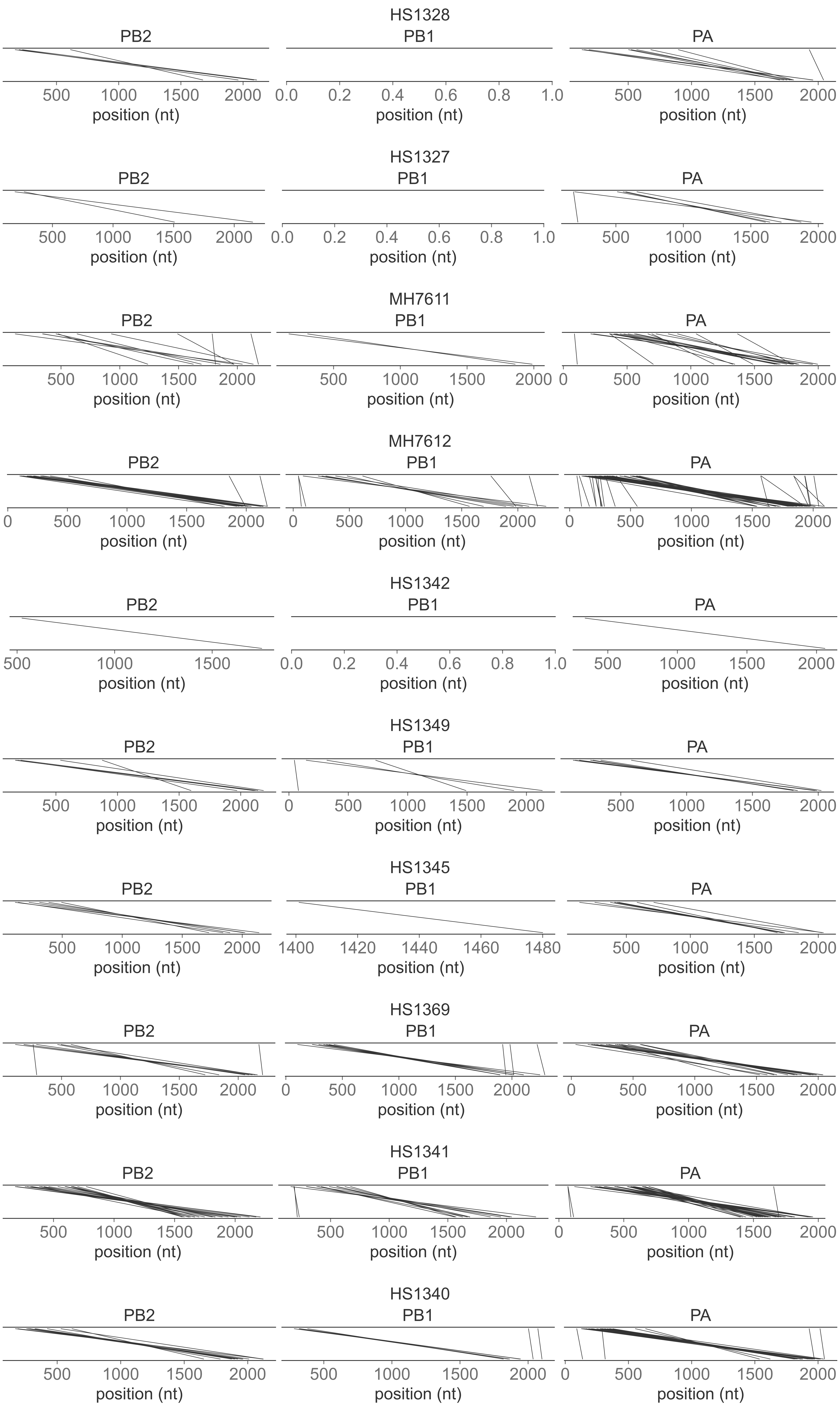

Figure S5 page 9

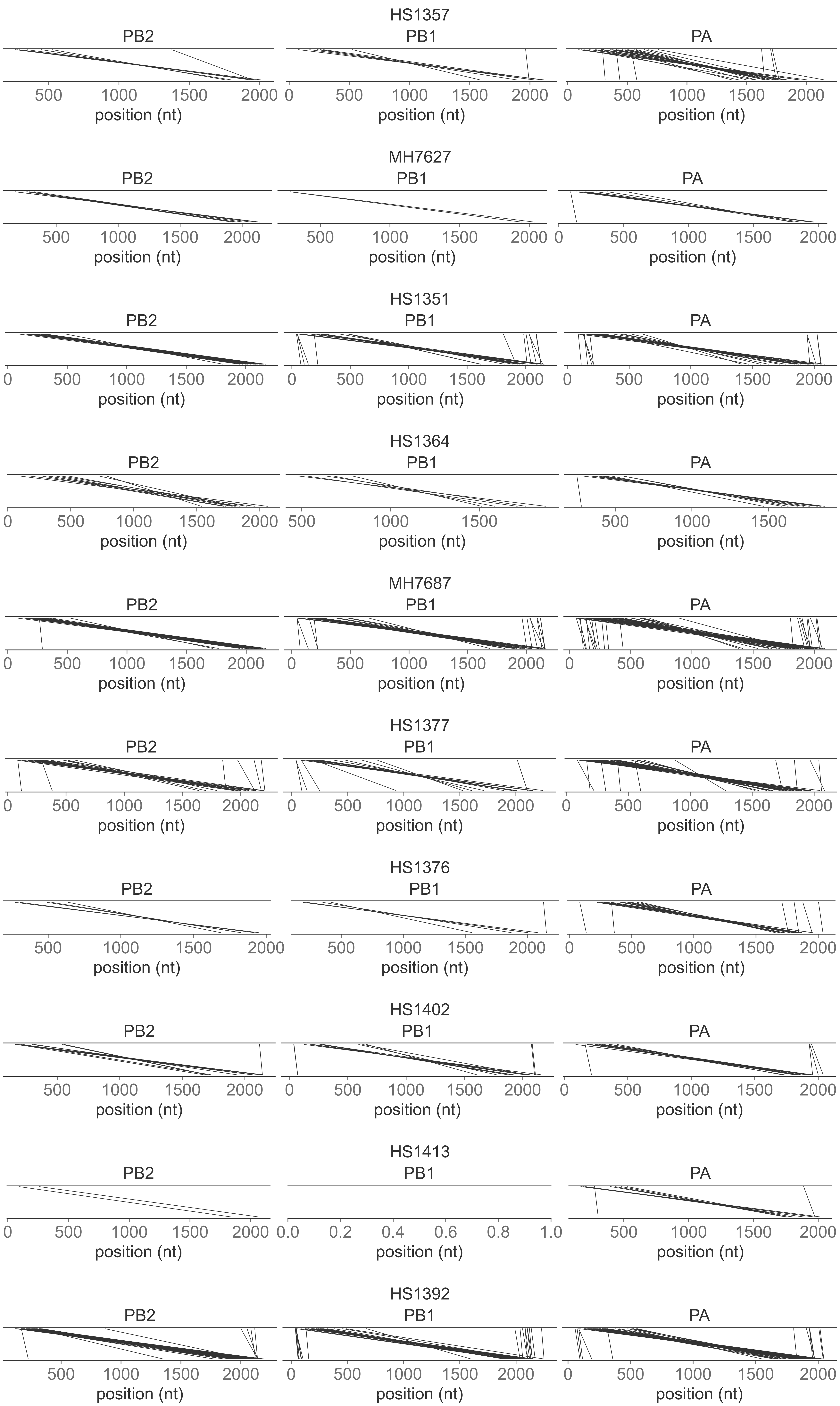

Figure S5 page 10

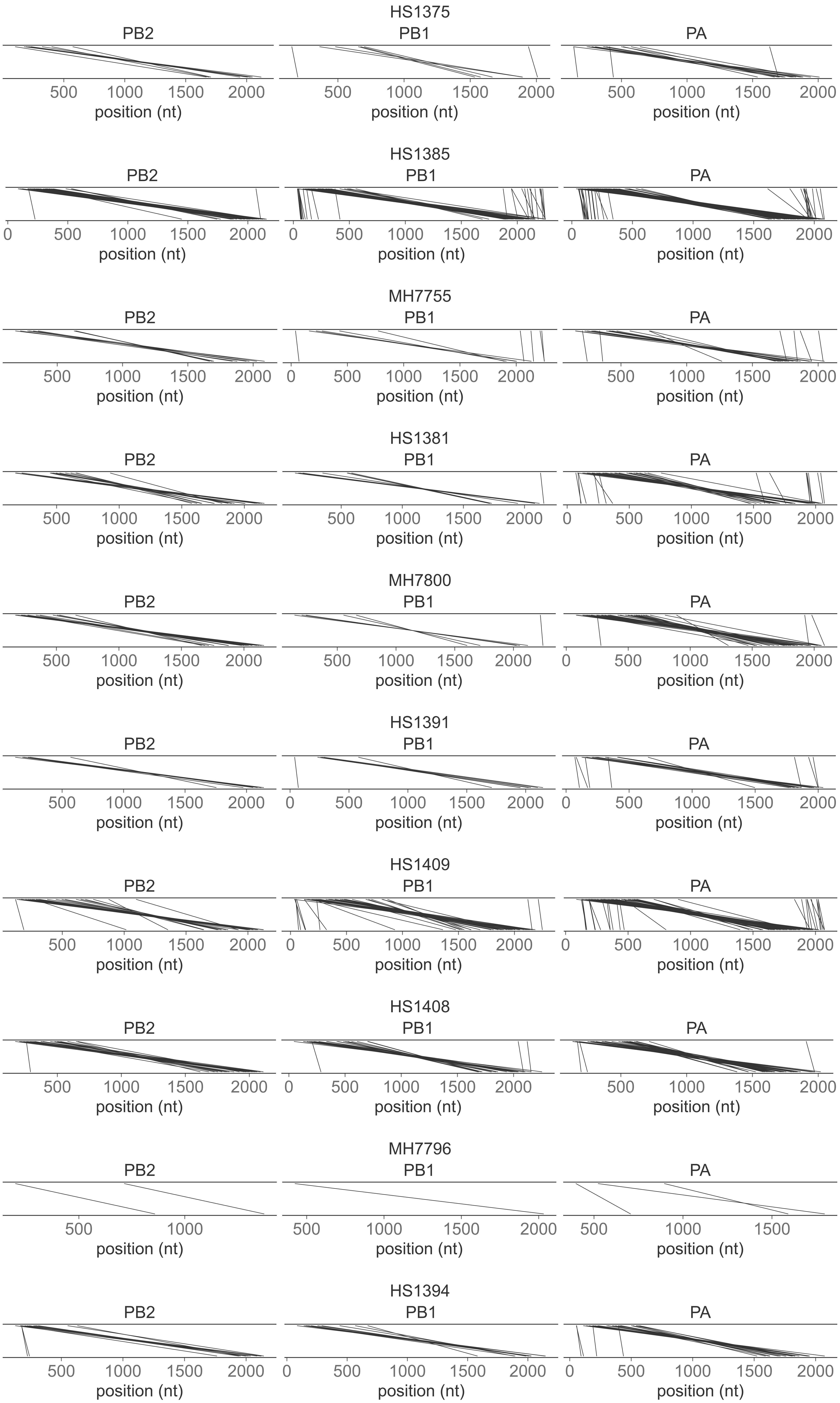

Figure S5 page 11

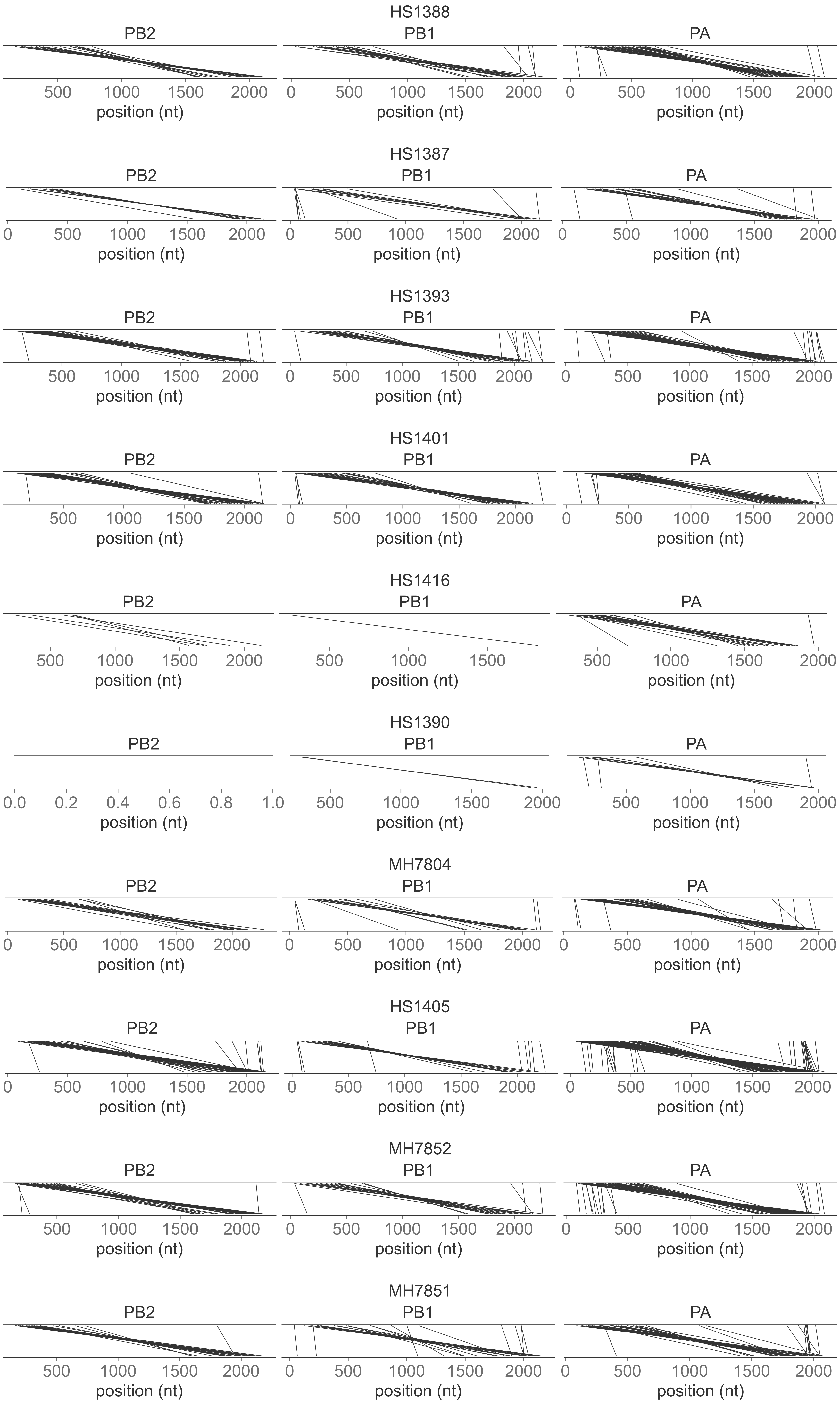

Figure S5 page 12

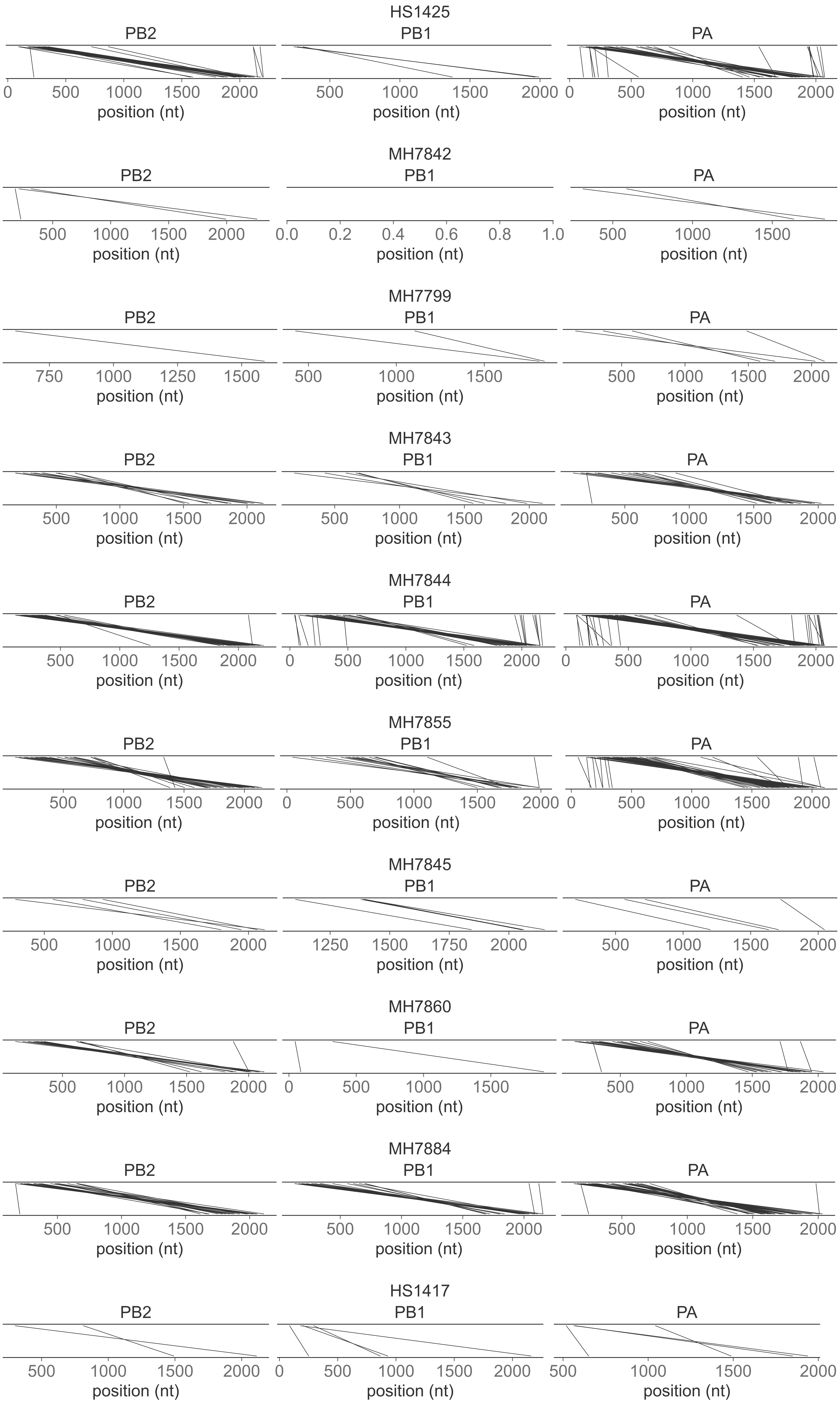

Figure S5 page 13

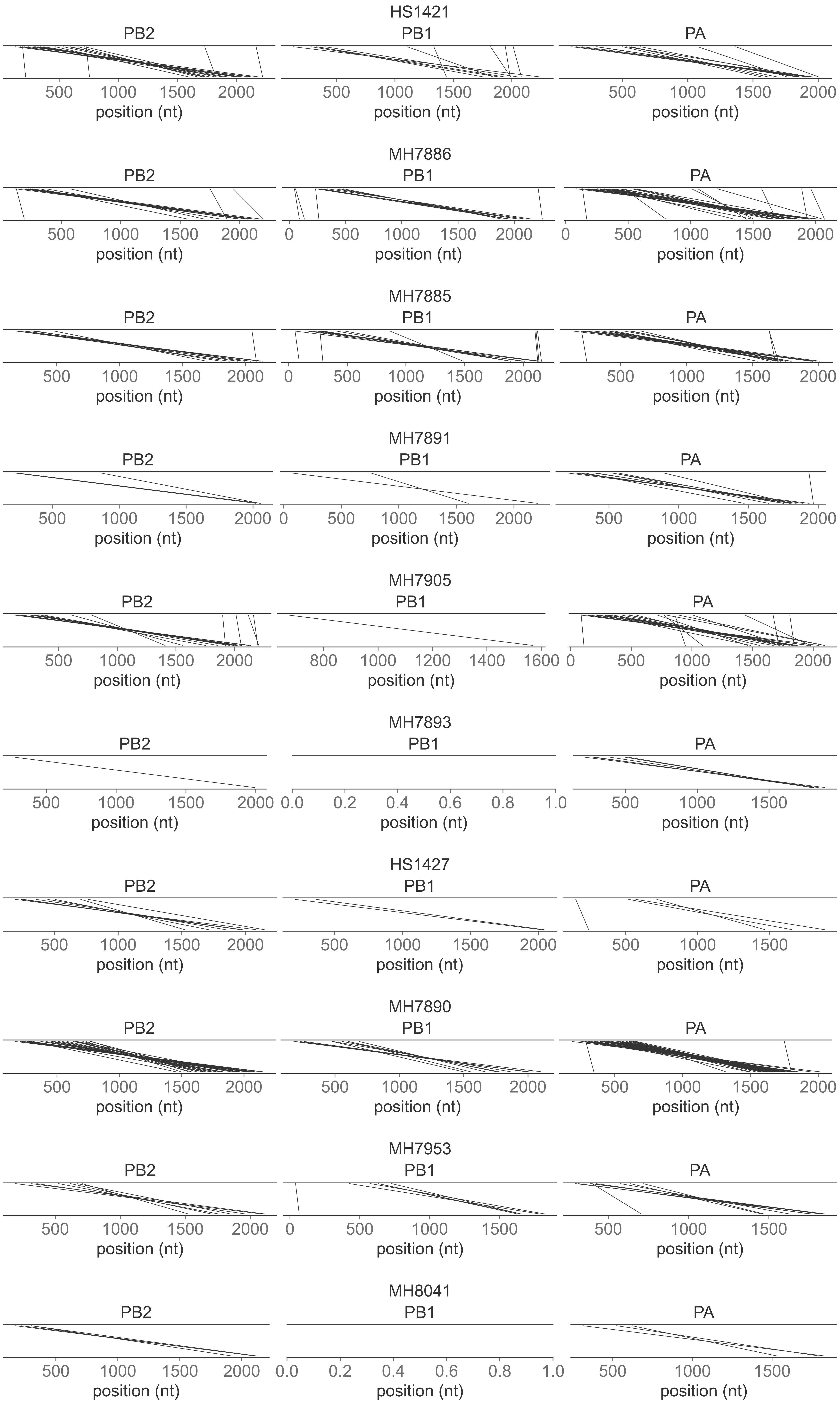

Figure S5 page 14

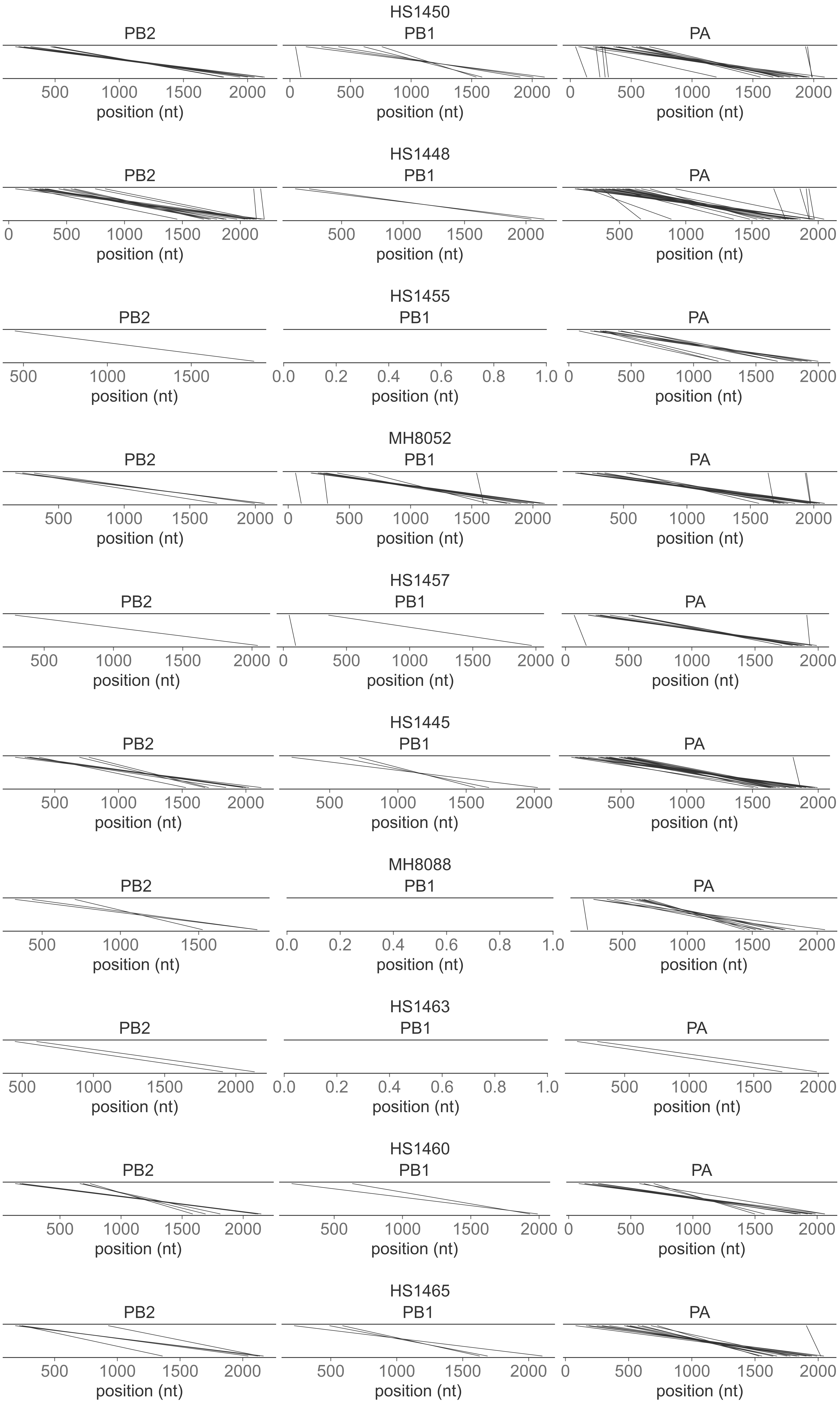

Figure S5 page 15

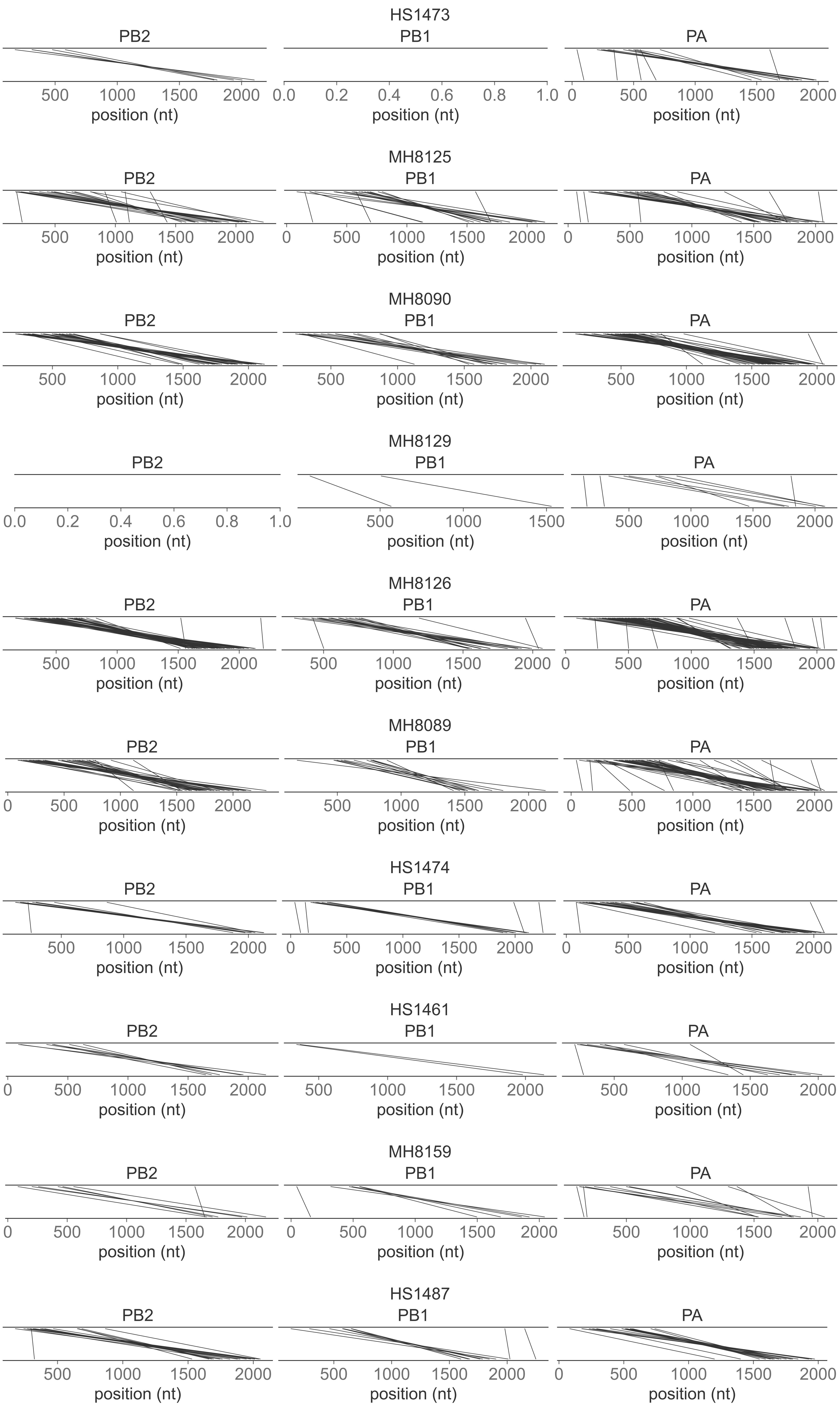

Figure S5 page 16

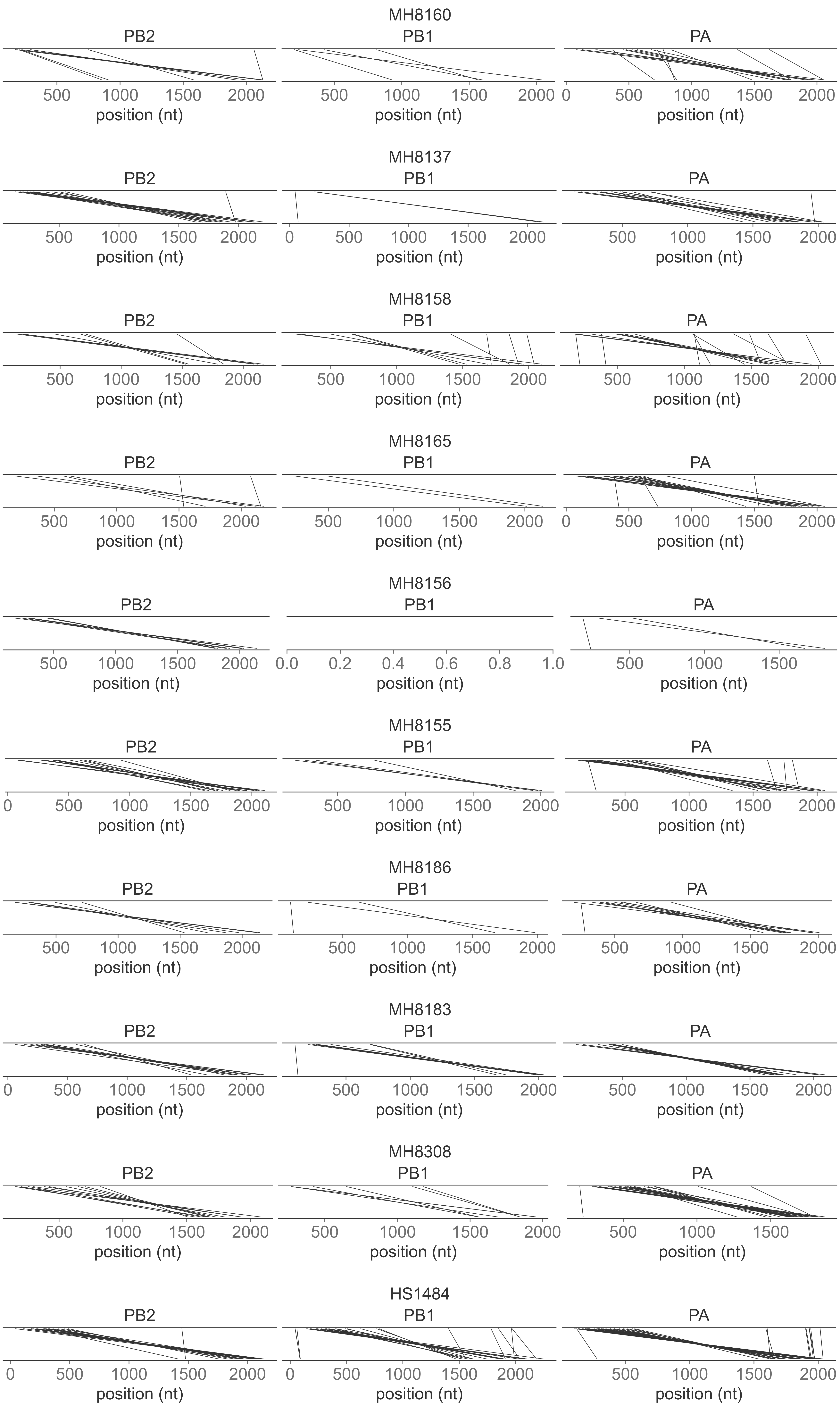

Figure S5 page 17

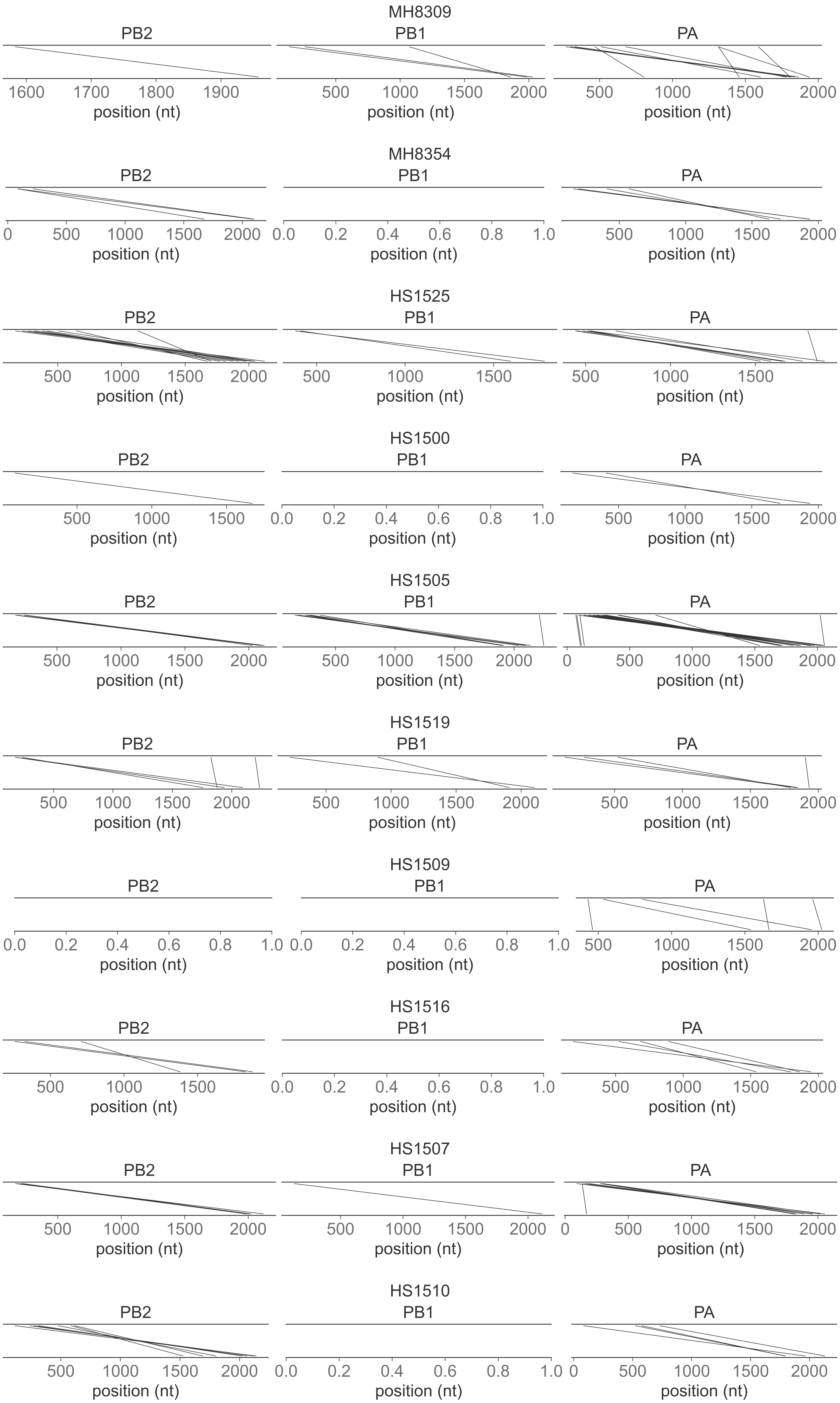

Figure S5 page 18

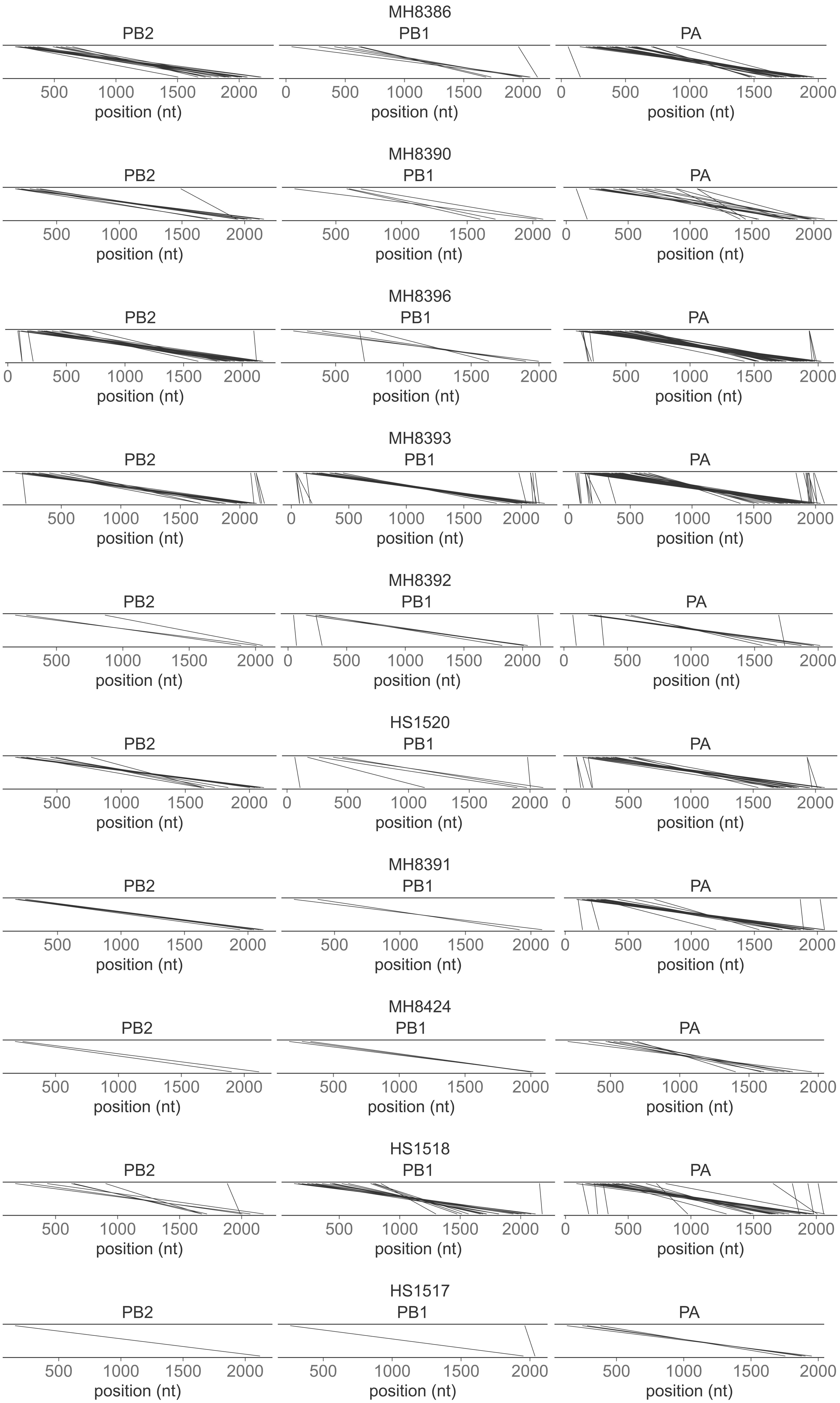

Figure S5 page 19

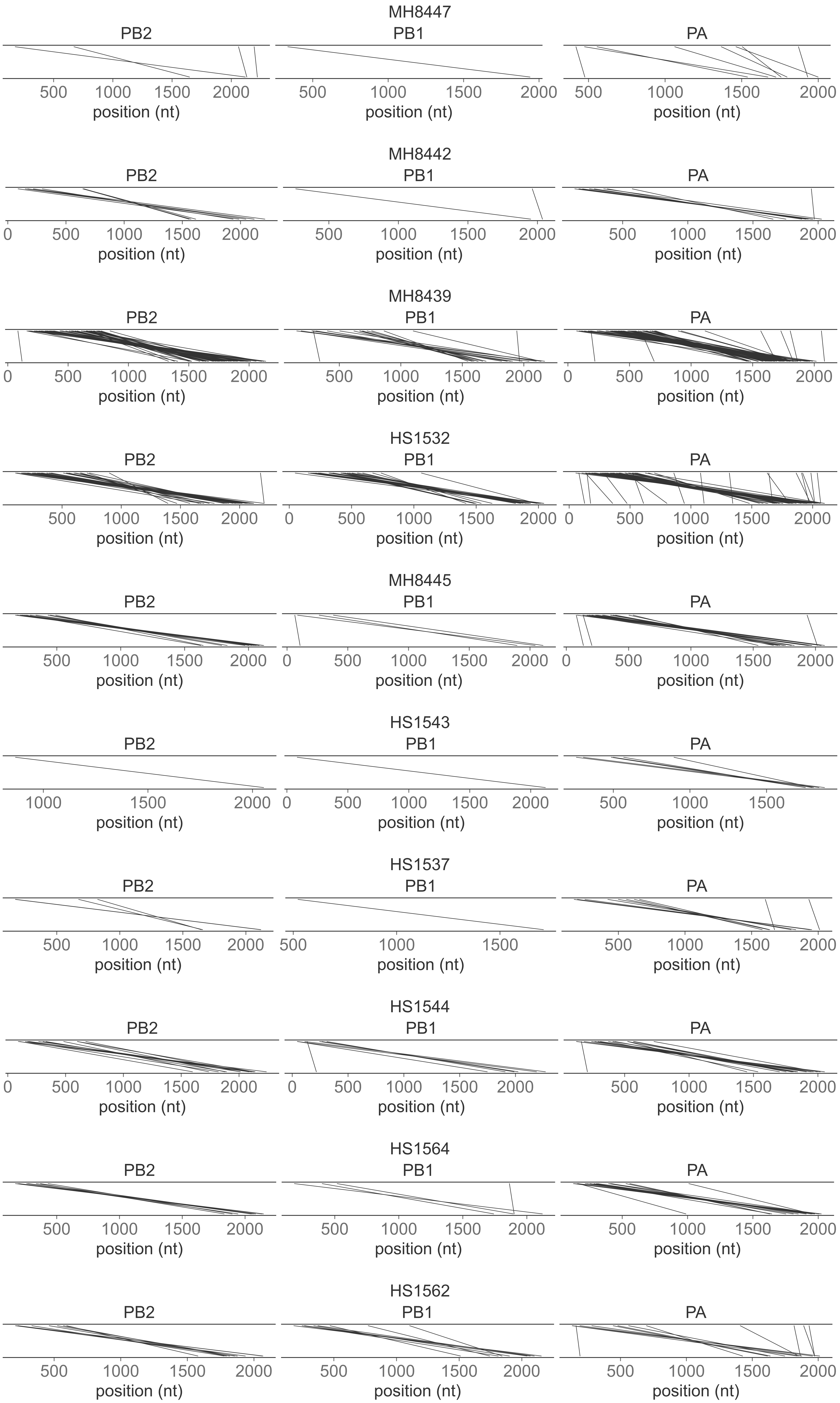

Figure S5 page 20

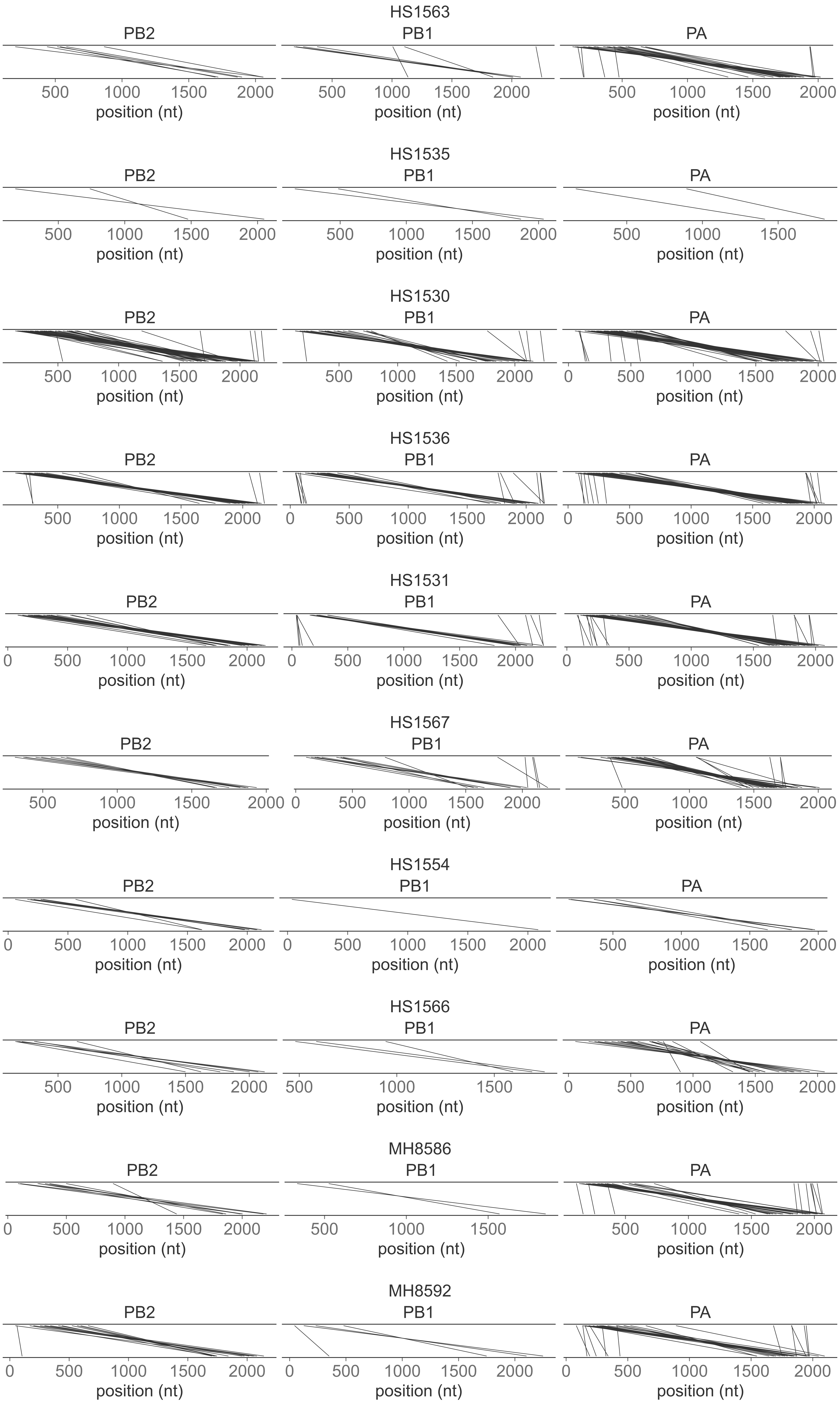

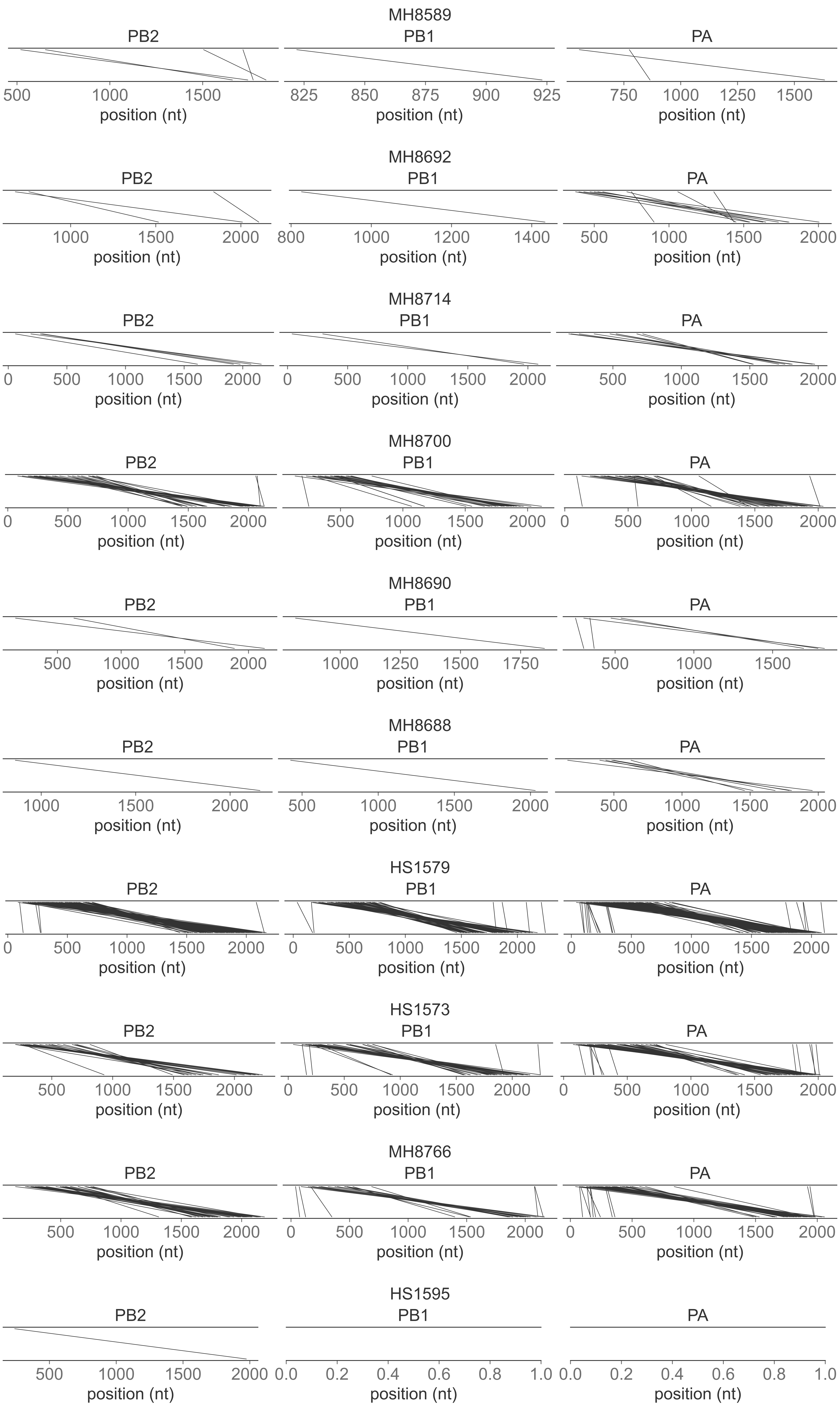
MH8589
PB2
PB1
PA
500 1000 1500
825 850 875 900 925
750 1000 1250 1500
position (nt)
MH8692
PB2
PB1
PA
1000 1500 2000
800 1000 1200 1400
500 1000 1500 2000
MH8714
MH8700
MH8690
500 1000 1500 2000
1000 1250 1500 1750
500 1000 1500
MH8688
HS1579
HS1573
MH8766
HS1595
0 500 1000 1500 2000
0.0 0.2 0.4 0.6 0.8 1.0
position (nt)

Figure S5 page 22

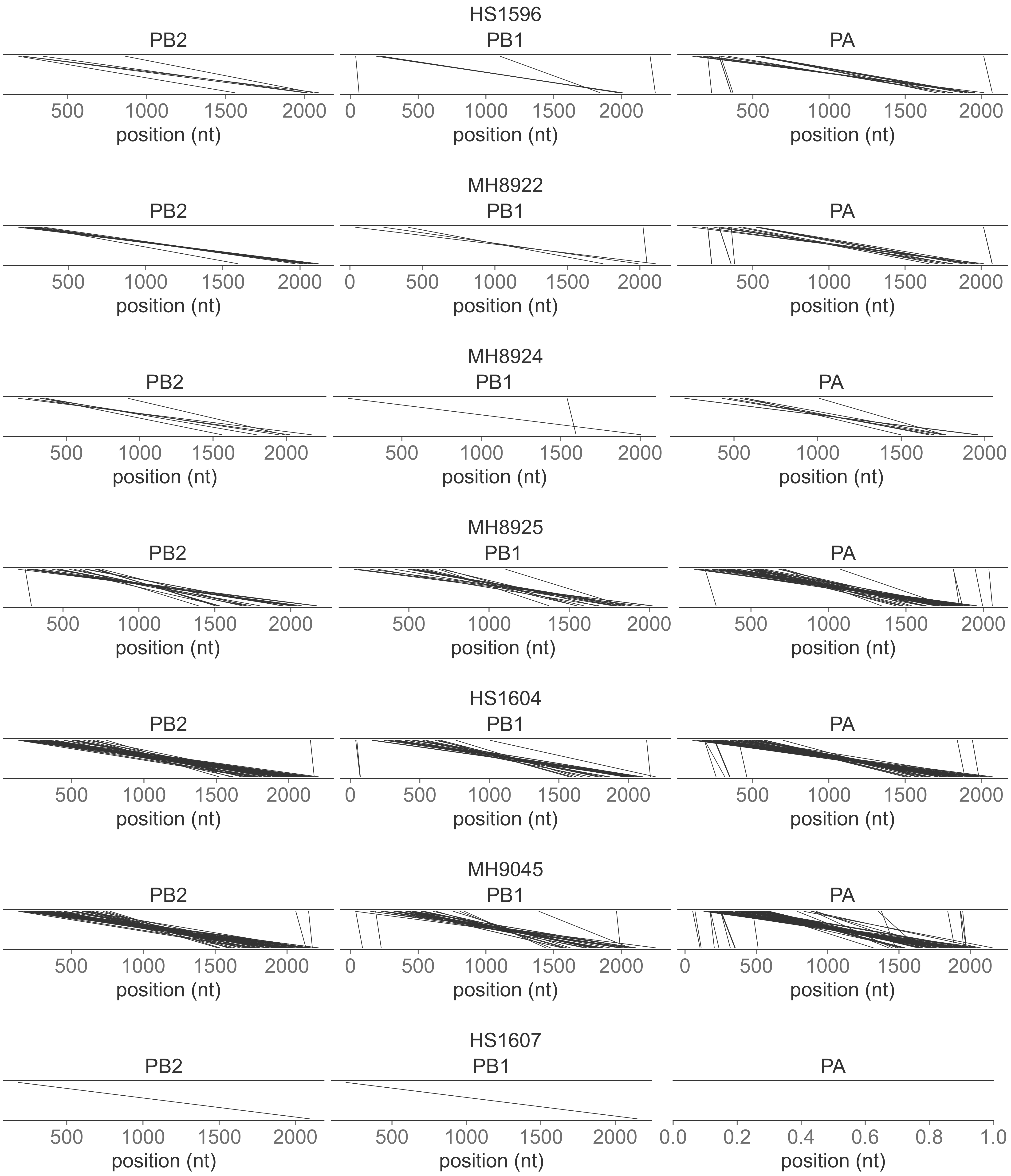

Supplementary Figure 5. DVG junction locations. Observed junction locations for all PB2, PB1, and PA DVGs observed in all clinical samples. Lines connect the nucleotides flanking the deleted nucleotides for each identified DVG.

Figure S6

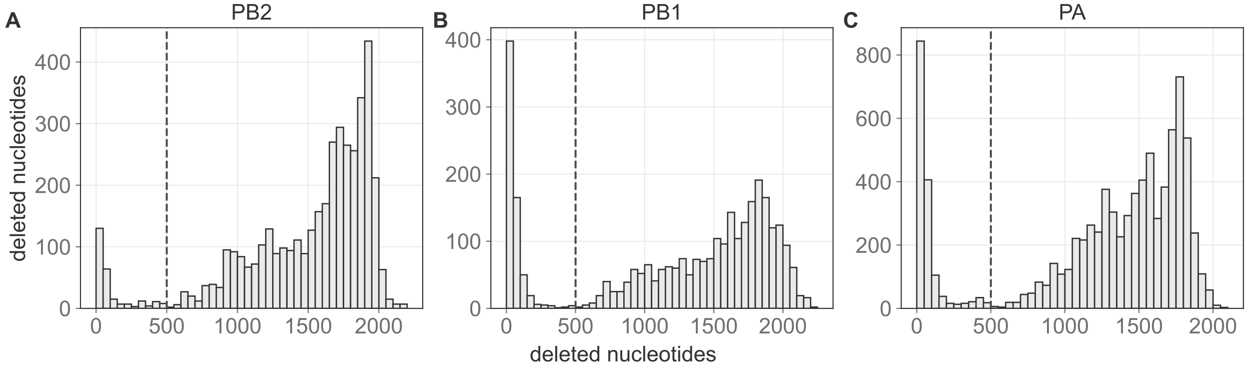

Supplementary Figure 6. Deleted nucleotides. Number of deleted nucleotides for each DVG identified in the PB2 (A), PB1 (B), and PA (C) segments. Dashed line at 500 nucleotides represents our empirical filtering threshold.

Figure S7 page 1

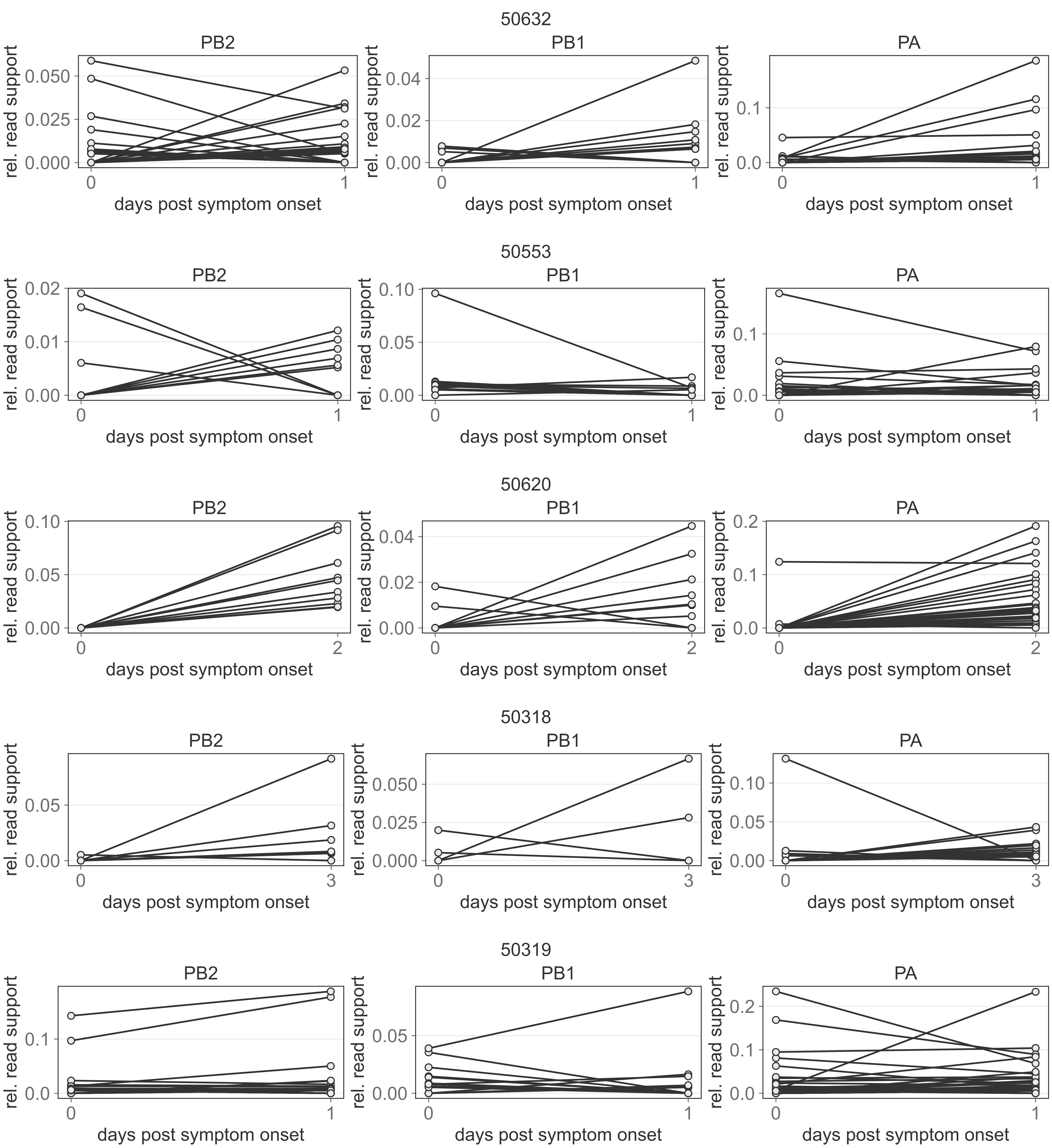

Figure S7 page 2

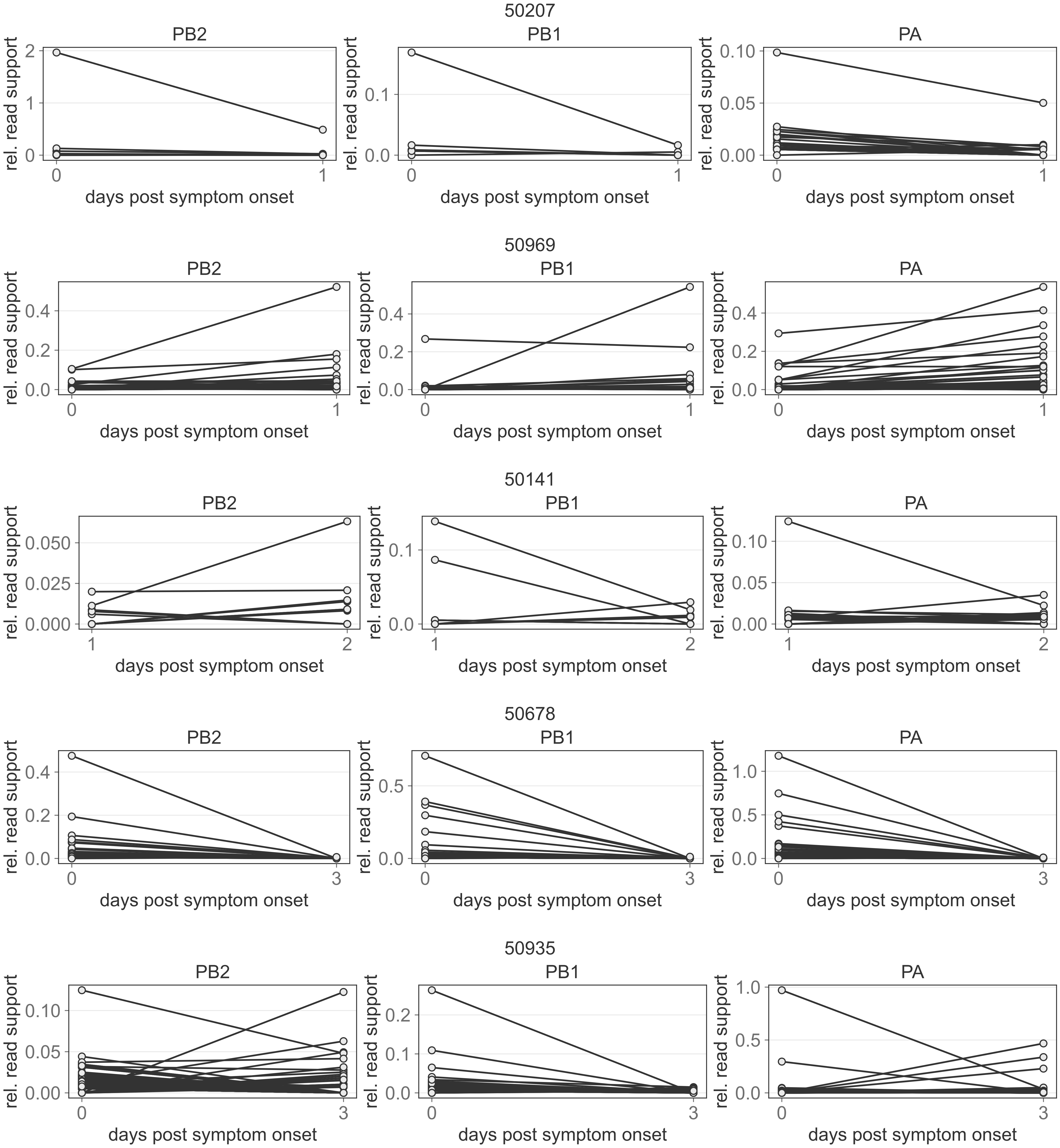

Figure S7 page 3

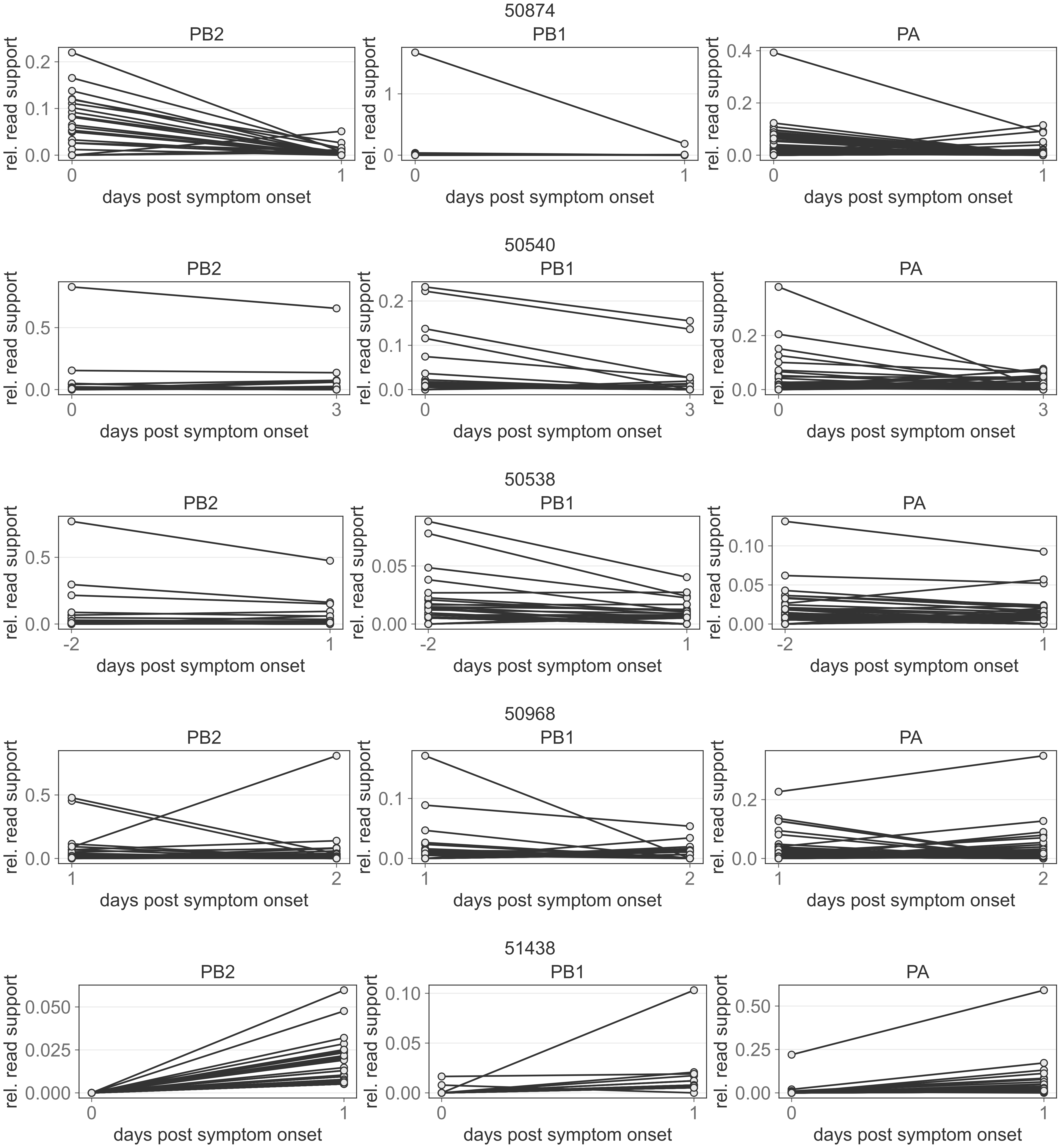

Figure S7 page 4

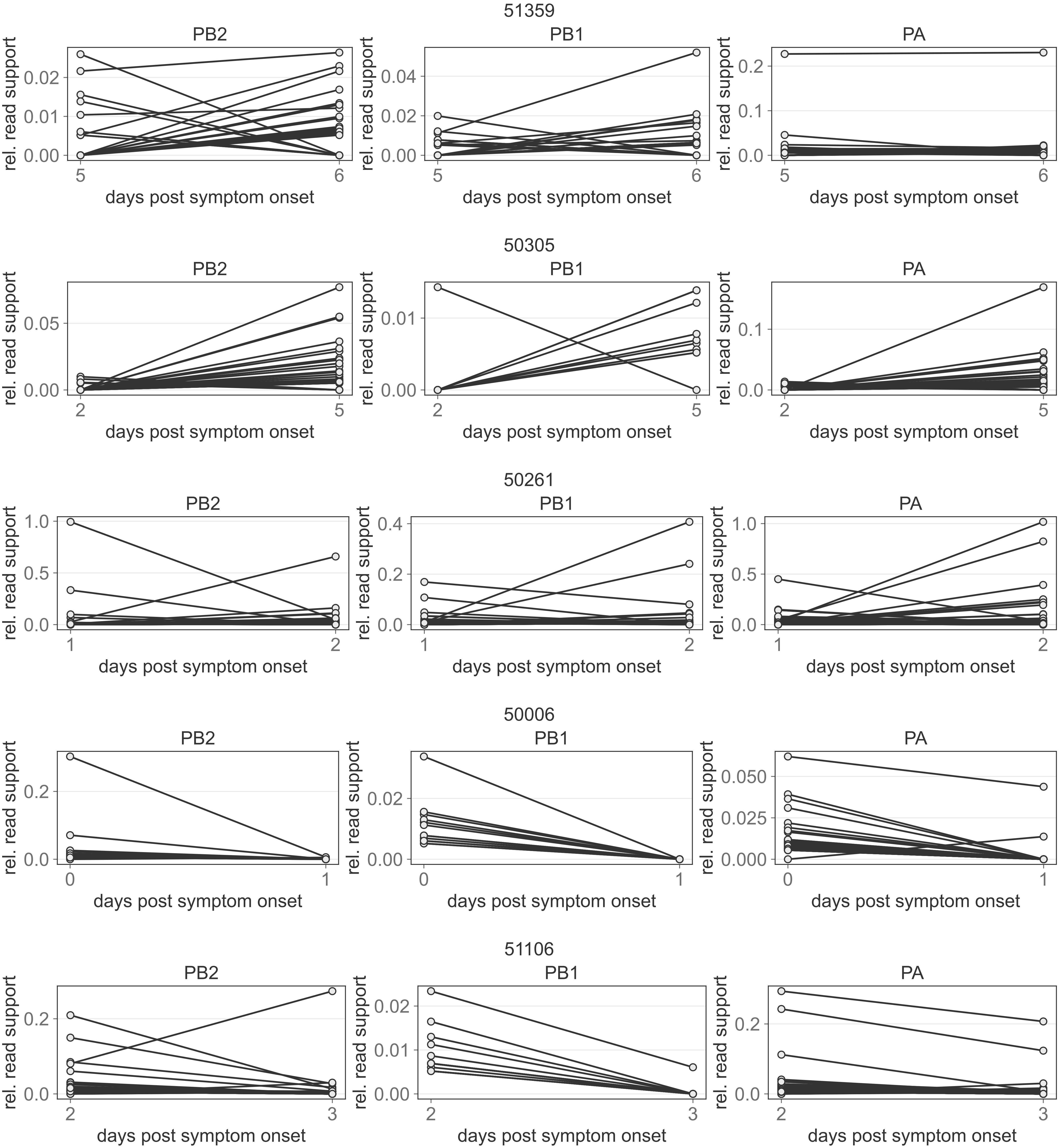

Figure S7 page 5

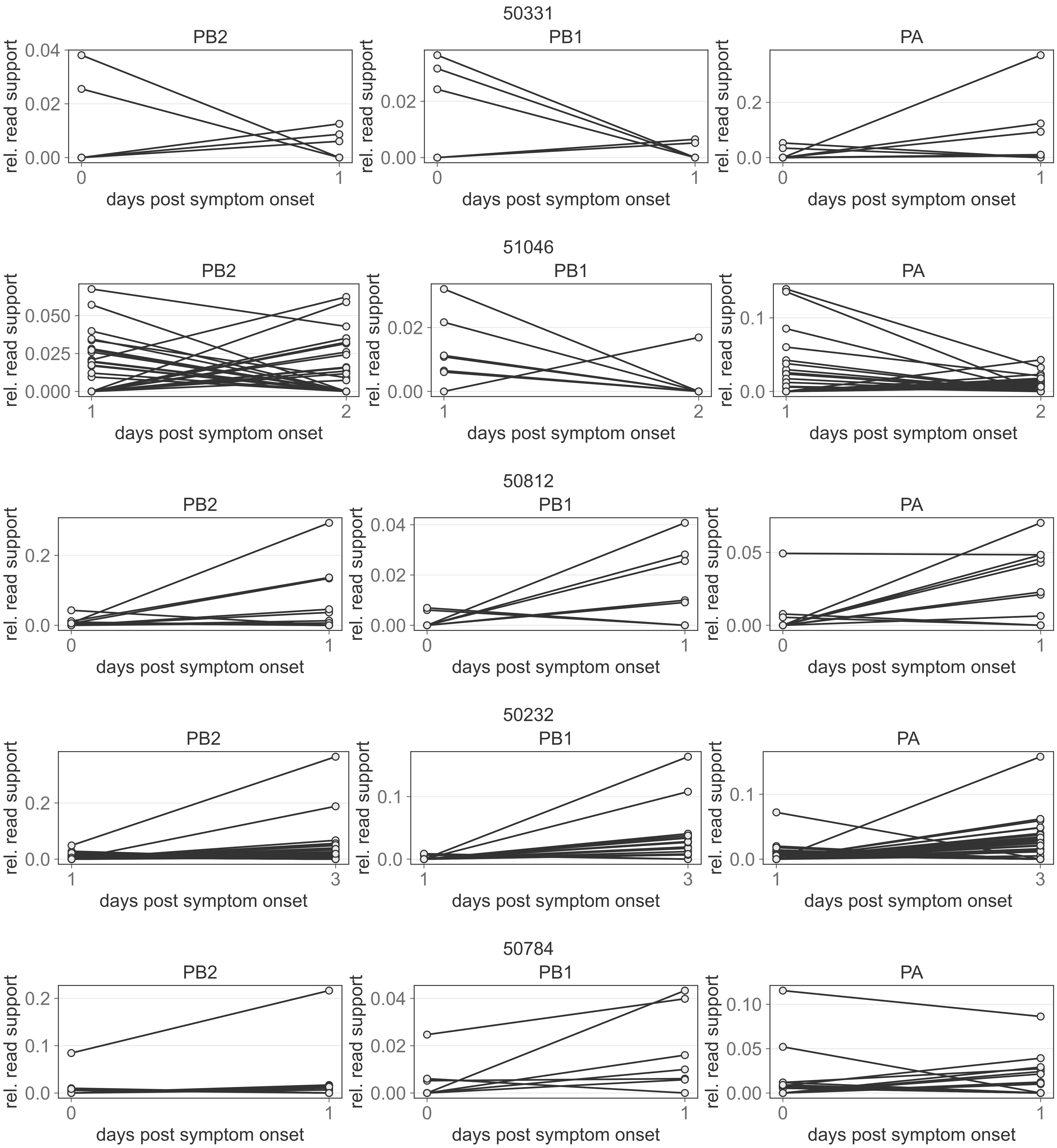

Figure S7 page 6

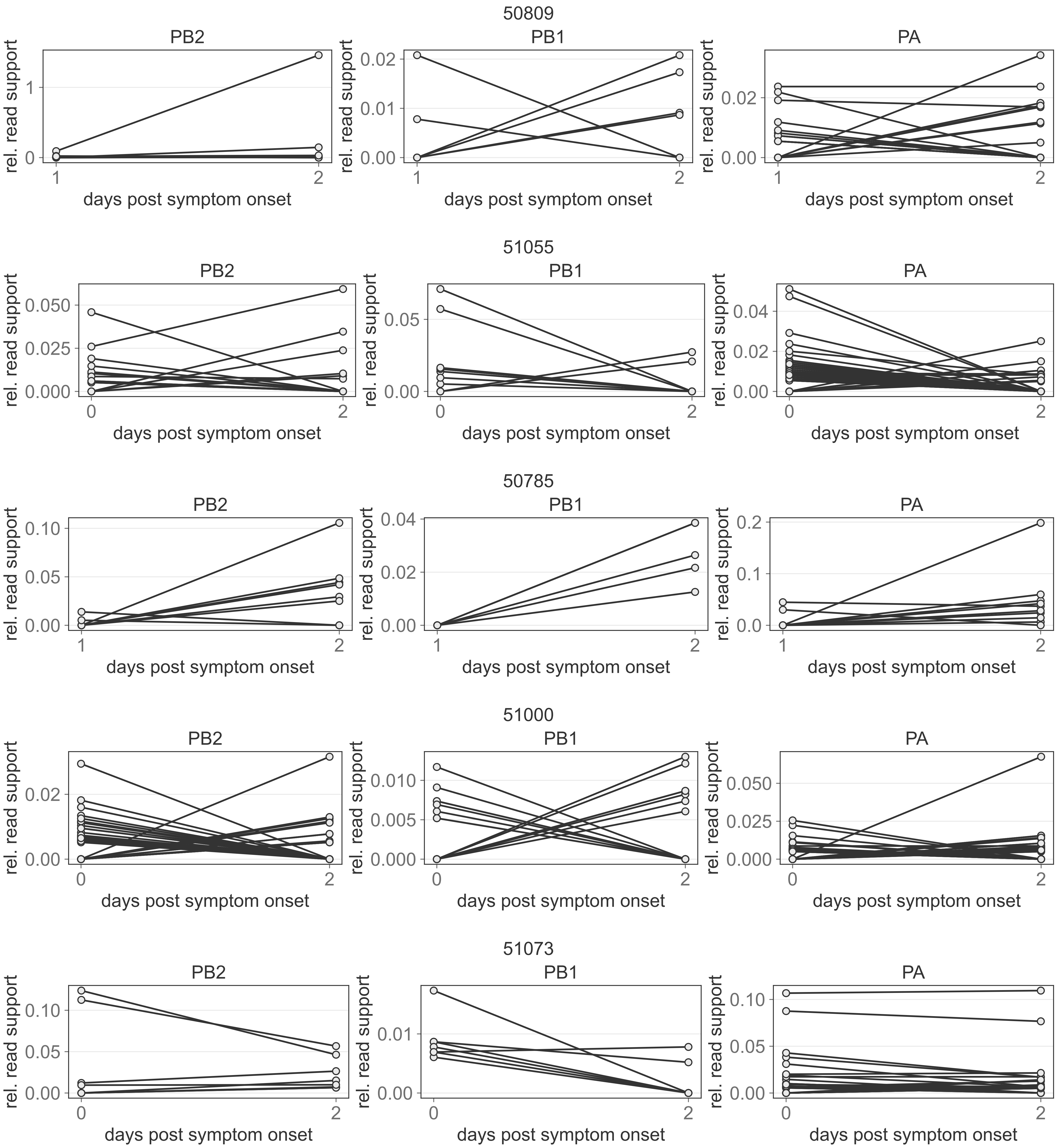

Figure S7 page 7

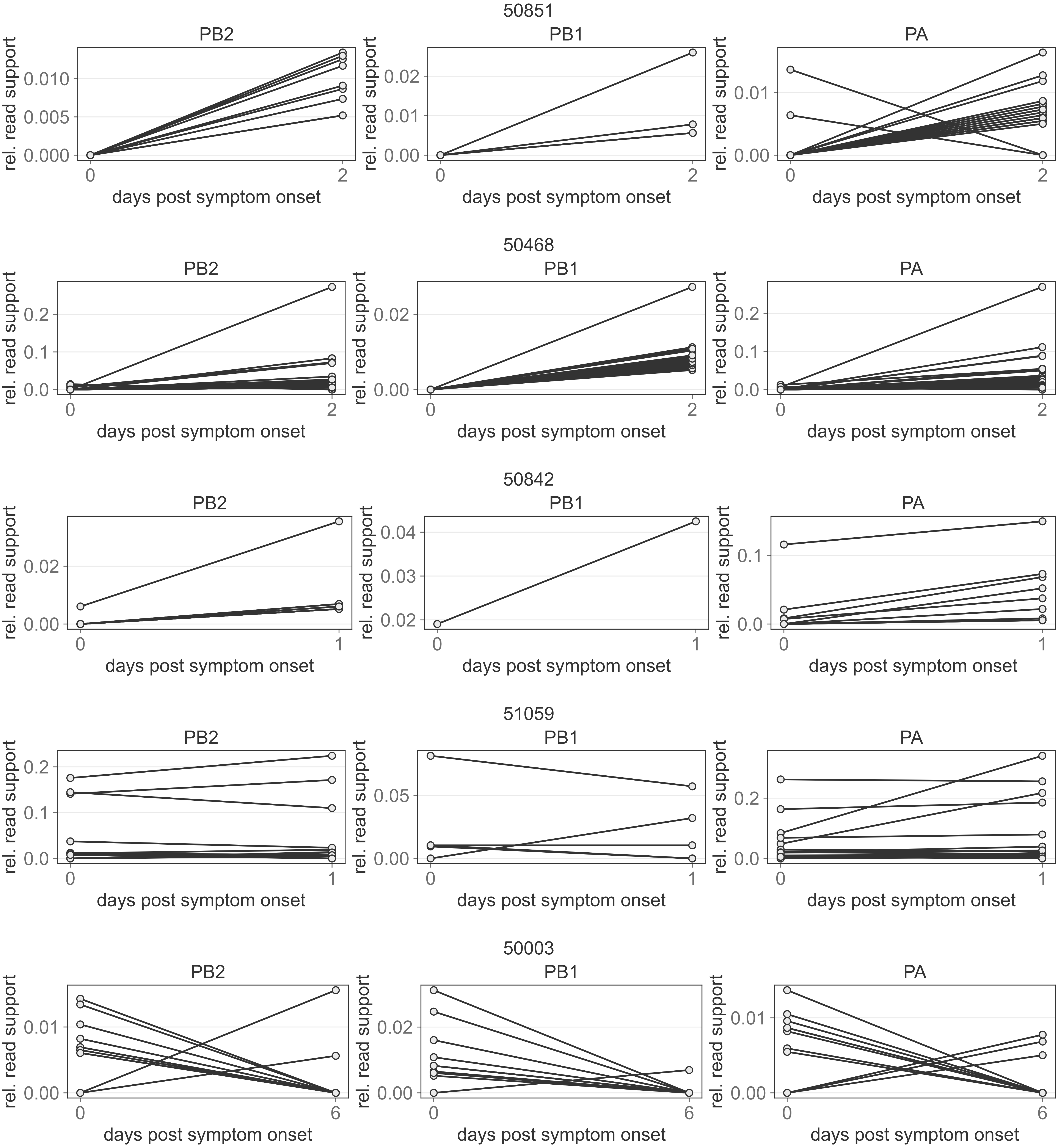

Figure S7 page 8

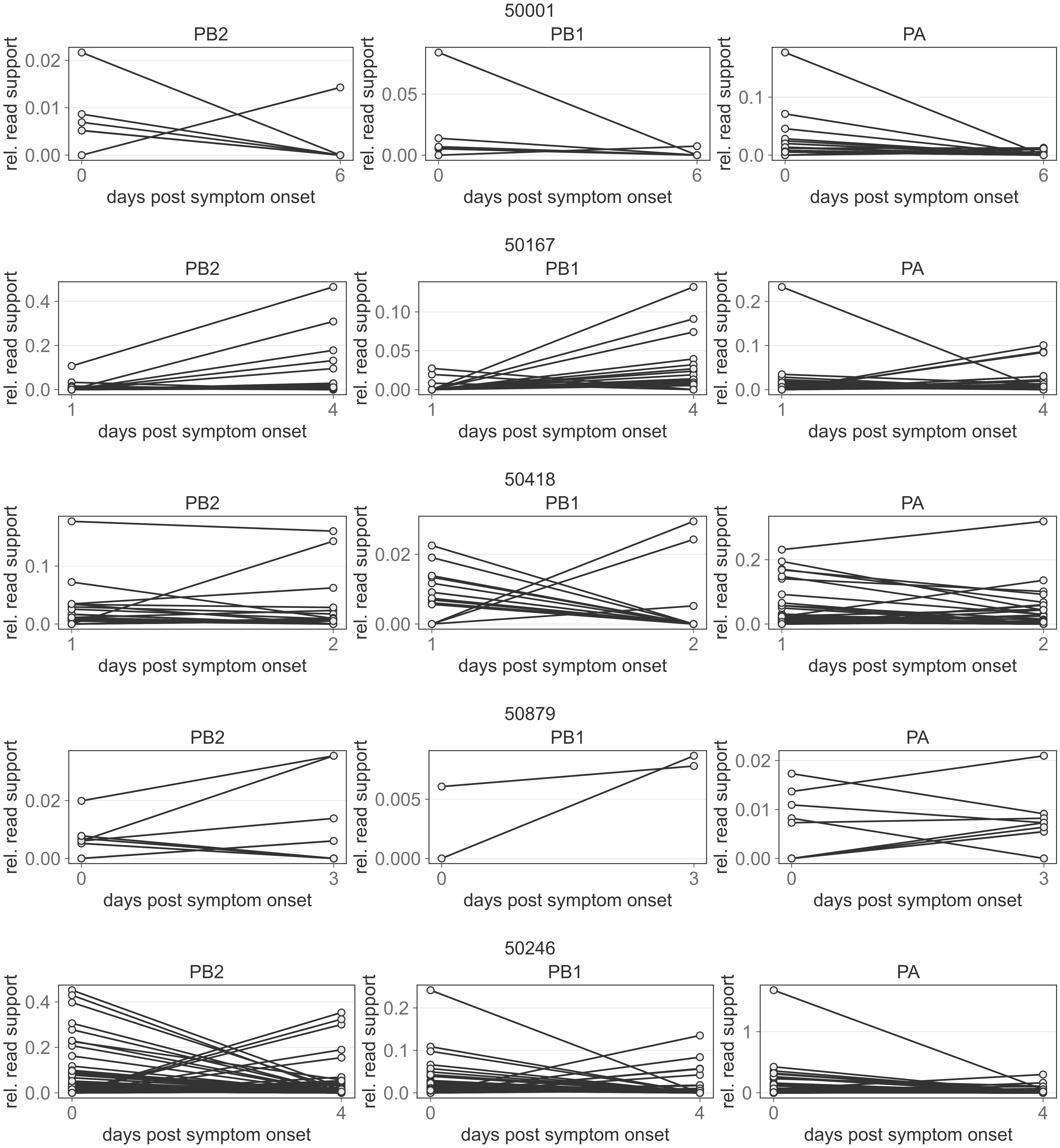

Figure S7 page 9

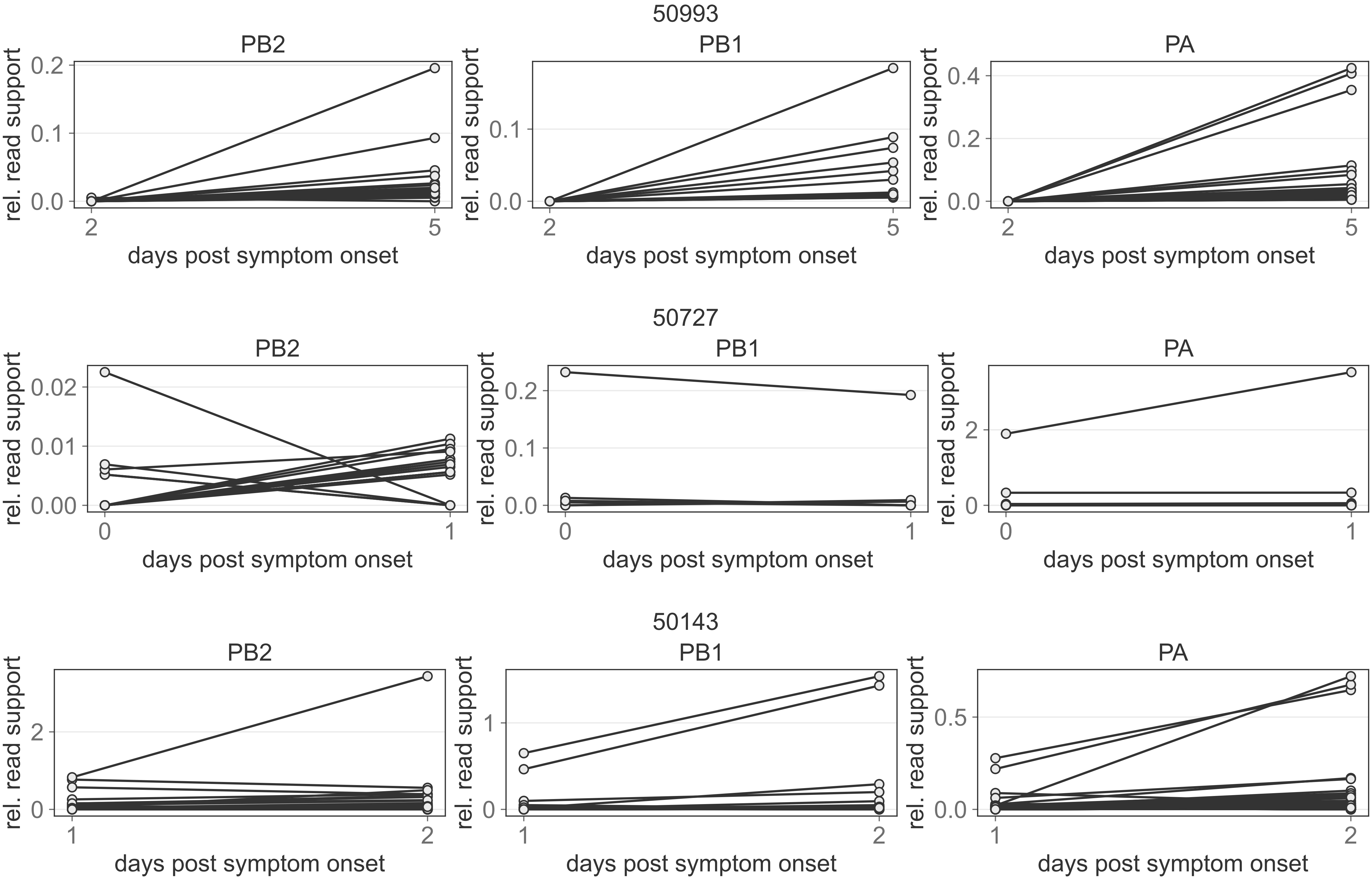

Supplementary Figure 7. Read support of longitudinal DVGs. Relative read support of DVGs identified in the PB2, PB1, and PA segments of 43 individuals with longitudinal samples taken at least one day apart.

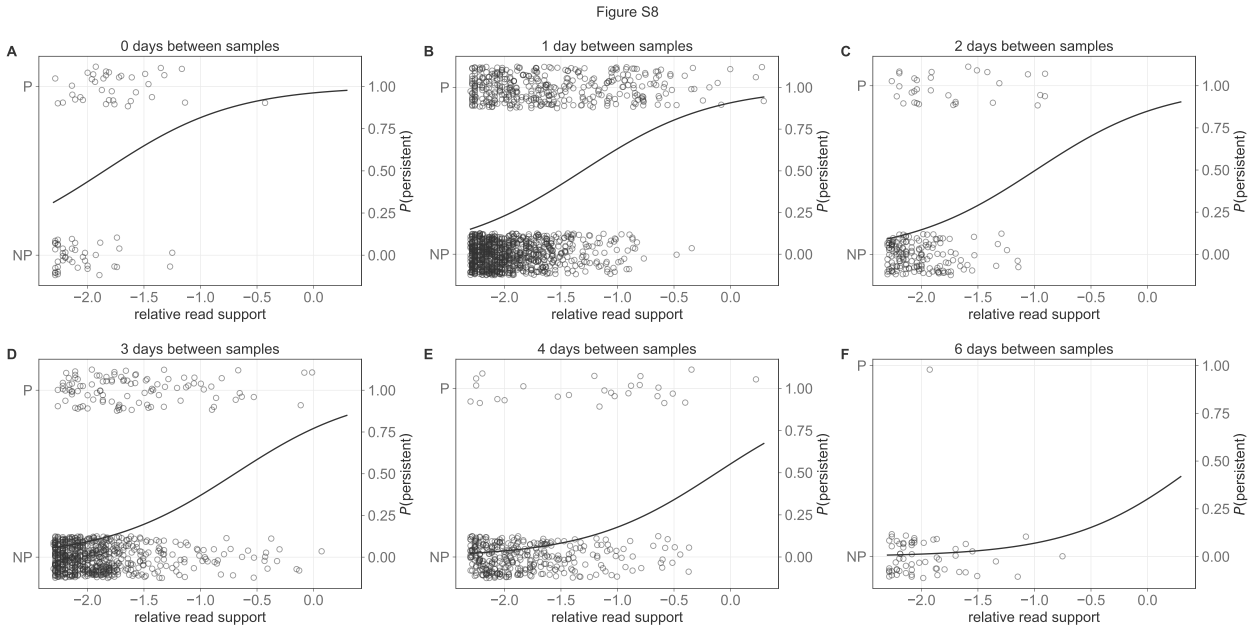

Supplementary Figure 8. Polymerase DVG persistence across time point and relative read support. Log_{10} relative read support of DVGs identified in longitudinal samples taken 0 (6 individuals), 1 (24 individuals), 2 (7 individuals), 3 (9 individuals), 4 (1 individual), and 6 (2 individuals) days apart, stratified by whether those DVGs are persistent (P) or non persistent (NP) between t_0 and t_1. Curve is the predicted probability of persistence from a multivariate logistic regression with $\log_{10}$ relative read support and time between samples (categorical) as predictors.

Figure S9

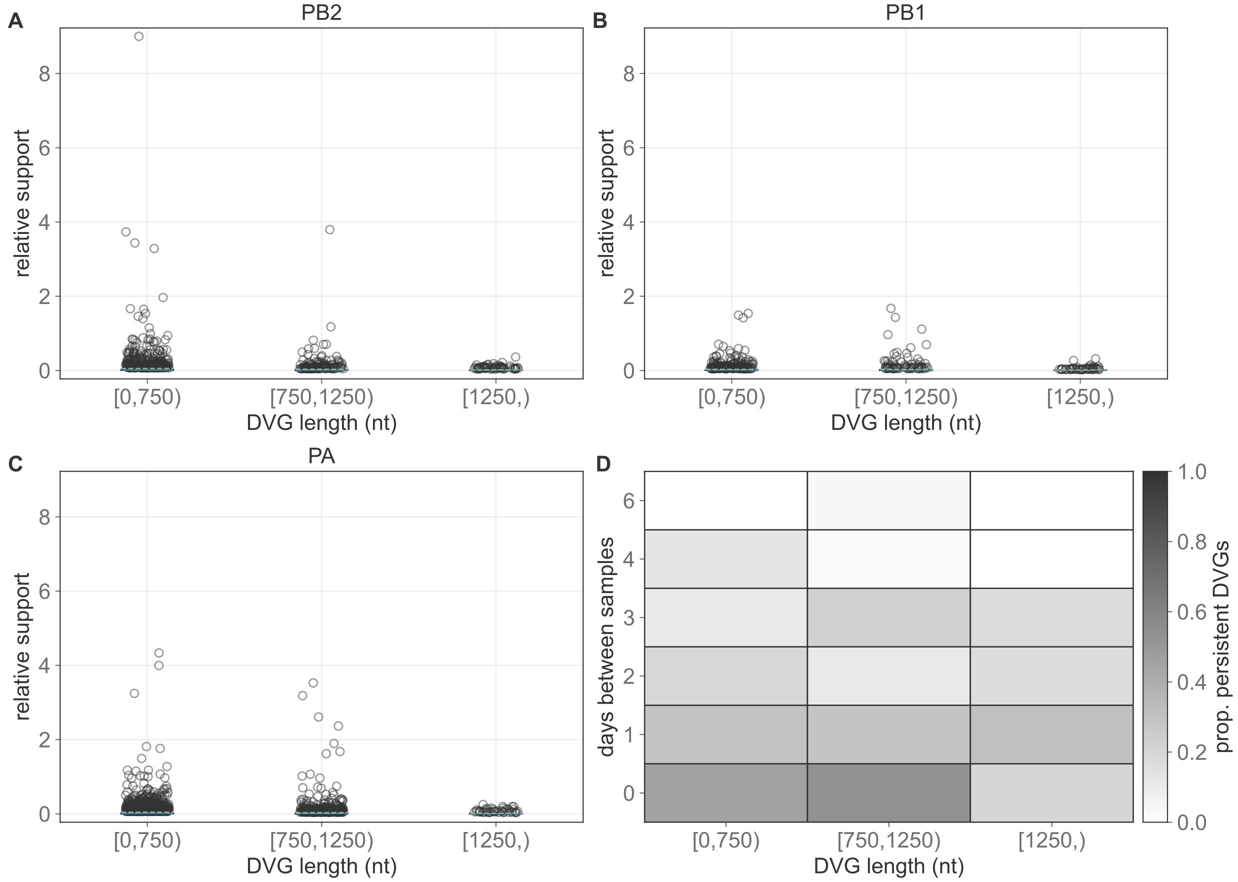

Supplementary Figure 9. Relative read support of polymerase DVGs stratified by length. Relative read support of DVGs identified in the PB2 (A), PB1 (B), and PA (C) segments stratified by their relative read support. In (A), (B), and (C), blue lines in the boxplots show the median value for each segment, dotted green lines show the mean, and box extends to the limits of the IQR, and whiskers extend to 1.5 IQR below and above the 1st and 3rd quartile, respectively. Outliers are shown as dots beyond the range of the whiskers. (D) Proportion of DVGs observed at the first time point for each of the 43 samples with longitudinal data that persistent to the second sampling time as a function of binned length and time between samples.

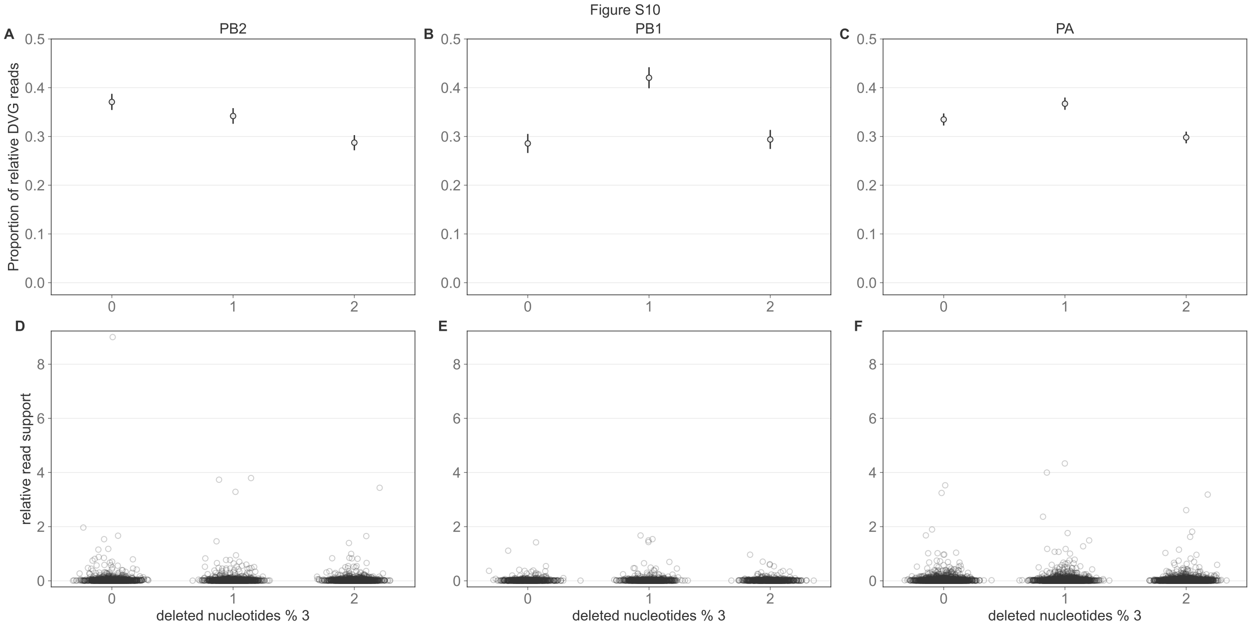

Supplementary Figure 10. Relative read support of polymerase DVGs stratified by deletion size modulus. Top row: proportion of polymerase DVG reads that support DVGs where the number of deleted nucleotides (D) mod (%) 3 = 0 (A), 1 (B), or 2 (C). Estimates are generated from 10,000 bootstrap samples of all observed DVGs weighted by their relative read support. Median estimate shown as a point, credibility bars extend from the 2.5th percentile to the 97.5th percentile. Bottom row: relative read support of DVGs identified in the PB2 (D), PB1 (E), and PA (F) segments stratified by their $D\%3$ value.

Segment	Variable	Coefficient	Std Err	p-value	0.025	0.975
PB2	Intercept	1.7314	0.489	0.000	0.767	2.696
PB2	$\log_{10}$ genomes/μL	-0.1880	0.103	0.071	-0.392	0.016
PB1	Interccept	0.6821	0.252	0.007	0.185	1.180
PB1	$\log_{10}$ genomes/μL	-0.0582	0.053	0.277	-0.163	0.047
PA	Intercept	2.3408	0.866	0.007	0.634	4.047
PA	$\log_{10}$ genomes/μL	-0.1813	0.183	0.323	-0.542	0.180
HA	Intercept	0.1266	0.053	0.018	0.022	0.231
HA	$\log_{10}$ genomes/μL	-0.0116	0.011	0.299	-0.034	0.010
NP	Intercept	0.1097	0.015	0.000	0.080	0.140
NP	$\log_{10}$ genomes/μL	-0.0158	0.003	0.000	-0.022	-0.009
NA	Intercept	0.2180	0.075	0.004	0.070	0.366
NA	$\log_{10}$ genomes/μL	-0.0226	0.016	0.155	-0.054	0.009
M	Intercept	0.3090	0.038	0.000	0.233	0.385
M	$\log_{10}$ genomes/μL	-0.0390	0.008	0.000	-0.055	-0.034
NS	Intercept	0.4658	0.099	0.000	0.270	0.661
NS	$\log_{10}$ genomes/μL	-0.0451	0.021	0.033	-0.086	-0.0004

Supplementary Table 1. Quantity of DVGs as a function of input titer. Linear regression of the total relative DVG reads in each sample as a function of the $\log_{10}$ genomes/μLmeasure for that sample.

Type	Sequence
NS 316_545 (CY207735.1 301_530)	ACAAAGACATAATGGATTCCAACACTGTGTCAAGT<br>TTCCAGGTAGATTGCTTTCTTTGGCATATCCGGAA<br>ACAAGTTGTAGACCAAAAACTGAGTGATGCCCCAT<br>TCCTCGATCGGCTTCGCCGAGATCAGAGGTCCCTA<br>AGGGGAAGAGGCAATACTCTCGGTCTAGACATCAA<br>AGCAGCCACCCATGTTGGAAAGCAAATTGTAGAAA<br>AGATTCTGAAAGAAGAATCTGATGAGGCACTTAAA<br>ATGACCATGGTCTCAACACCTGCTTCGCGATACAT<br>AACTGACATGACTATTGAGGATGTCAAAAATGCAA<br>TTGGGGTCCTCATCGGAGGACTTGAATGGAATGAT<br>AACACAGTTCGAGTCTCTAAAAATCTACAGAGATT<br>CGCTTGGAGAAGCAGTAATGAGAATGGGGGACCTC<br>CACTTACTCCAAAACAGAAACGGGAAATGGCGAGA<br>ACAGCTAGGTCAGAAGTTTGAAGAGATAAGATGGC<br>TAATTGAAGAGGTGAGACACAGATTAAGAACAACT<br>GAAAATAGCTTTGAACAAATAACATTCATGCAAGC<br>ATTACAACTACTGTTTGAAGTGGAACAGGAGATAA<br>GAACTTTCTCATTTCAGCTTATTTAATGATAAAAAAC
NS 316_545 supporting read (SRR6121205 1_26672)	CGCGATACATAACTGACATGACTATTGAGGATGTC<br>AAAAATGCAATTGGGGTCCTCATCGGAGGACTTGA<br>ATGGAATGATAACACAGTTCGAGTCTCTAAAAATC<br>TACAGAGATTCGCTTGGAGA

Supplementary Table 2. DVG NS 316_545 sequence. Nucleotide sequence of the NS segment (CY207735.1) with the 316_545 deletion and a representative supporting read. Deletion coordinates are given in our universal numbering system and correspond to 301_530 in segment CY207735.1.

Supplementary Table 3. DVG NS 316_545 segment BLASTN. BLASTN output of an NS segment sequence (CY207735.1) with the 316_545 deletion. Deletion coordinates are given in our universal number system and correspond to 301_530 in CY207735.1.

Supplementary Table 4. DVG NS 316_545 read BLASTN. BLASTN output of a representative read supporting the NS (CY207735.1) 316_545 deletion. Deletion coordinates are given in our universal number system and correspond to 301_530 in CY207735.1. Read derrived from sequencing run SRR6121205.

Variable	Coefficient	Std Err	p-value	0.025	0.975
Intercept	2.3434	0.281	0.000	1.7921	1.895
$\log_{10}$ relative support	1.7786	0.153	0.000	1.479	2.078

Supplementary Table 5. Polymerase DVG persistence across time points. Logistic Regression of the probability of DVG persistence between t_0 and t_1 as a function of $\log_{10}$ relative DVG read support.

Variable	Coefficient	Std Err	p value	0.025	0.975
Intercept	3.2334	0.322	0.000	2.602	3.865
C($t_{span} = 1$)	-0.9452	0.243	0.000	-1.421	-0.470
C($t_{span} = 2$)	-1.5056	0.308	0.000	-2.110	-0.901
C($t_{span} = 3$)	-2.0168	0.261	0.000	-2.528	-1.506
C($t_{span} = 4$)	-3.02342	0.323	0.000	-3.656	-2.391
C($t_{span} = 6$)	-4.0744	1.042	0.000	-6.117	-2.032
$\log_{10}$ relative support	1.7478	0.114	0.000	1.524	1.972

Supplementary Table 6. Polymerase DVG persistence across time point and relative read support. Logistic Regression of the probability of DVG persistence between t_0 and t_1 as a function of time between samples and $\log_{10}$ relative DVG read support.

Variable	Coefficient	Std Err	p value	0.025	0.975
Intercept	3.1524	0.325	0.000	2.516	3.789
C($t_{span}=1$)	-0.8934	0.245	0.000	-1.373	-0.414
C($t_{span}=2$)	-1.4714	0.309	0.000	-2.077	-0.866
C($t_{span}=3$)	-1.9442	0.263	0.000	-2.460	-1.428
C($t_{span}=4$)	-3.0257	0.323	0.000	-3.660	-2.392
C($t_{span}=6$)	-4.0650	1.042	0.000	-6.107	-2.032
C(DVG length = [750,120))	0.2606	0.115	0.024	0.034	0.487
C(DVG length = [1250,))	0.1929	0.216	0.371	-0.230	0.616
$\log_{10}$ relative support	1.78048	0.116	0.000	1.554	2.007

Supplementary Table 7. Polymerase DVG persistence across time point, relative read support, and DVG length. Logistic regression of the probability of DVG persistence between t_0 and t_1 as a function of time between samples, $\log_{10}$ relative DVG read support, and binned DVG length ([0, 750), [750,1250), [1250,) nucleotides.

Variable	Coefficient	Std Err	p value	0.025	0.975
Intercept	3.0998	0.344	0.000	2.425	3.774
C($t_{span} = 1$)	-0.9223	0.244	0.000	-1.400	-0.445
C($t_{span} = 2$)	-1.4888	0.309	0.000	-2.094	-0.884
C($t_{span} = 3$)	-1.9835	0.263	0.000	-2.498	-1.469
C($t_{span} = 4$)	-3.0228	0.323	0.000	-3.655	-2.390
C($t_{span} = 6$)	-4.0682	1.042	0.000	-6.110	-2.027
$\log_{10}$ relative support	1.7608	0.115	0.000	1.535	1.986
DVG length	0.0002	0.000	0.274	-0.000	0.001

Supplementary Table 8. Polymerase DVG persistence across time point, relative read support, and DVG length. Logistic Regression of the probability of DVG persistence between t_0 and t_1 as a function of time between samples, $\log_{10}$ relative DVG read support, and continuous DVG length.
